# The effect and mechanism of iodophors on the adhesion and virulence of *Staphylococcus aureus* biofilms attached to artificial joint materials

**DOI:** 10.1186/s13018-023-04246-x

**Published:** 2023-10-05

**Authors:** Sihui Chen, Yi Jiang, Wei Wang, Junjie Chen, Jinyu Zhu

**Affiliations:** 1 Department of Orthopaedics, First Hospital of Jiaxing, South Central Avenue No. 1882, Jiaxing, 314000 People’s Republic of China; 2 College of Medicine, Jiaxing University, Guangqiong Avenue No. 899, Jiaxing, 314000 People’s Republic of China; 3 Department of Clinical Laboratory, First Hospital of Jiaxing, South Central Avenue No. 1882, Jiaxing, 314000 People’s Republic of China; 4 Zhejiang Chinese Medical University Master Degree Cultivation Base in Jiaxing University, South Central Avenue No. 1882, Jiaxing, 314000 People’s Republic of China

**Keywords:** Iodophor, Biofilm, *Staphylococcus aureus*, Artificial joint materials, Adhesion, Virulence

## Abstract

**Background:**

Iodophors are known to be a treatment for biofilm-related periprosthetic joint infection. However, the efficacy and mechanism of eradicating biofilms from different artificial joint materials after iodophor treatment are unknown. This study was conducted to understand the effect and mechanism of iodophors with respect to the adhesion and virulence of *Staphylococcus aureus* biofilms attached to artificial joint materials.

**Methods:**

Biofilms of *Staphylococcus aureus* strains were grown on titanium alloy, cobalt chromium molybdenum and polyethylene coupons, which are commonly used materials for artificial joints, for 24 h. Afterward, all coupons were divided into experimental and control groups: (1) exposed to a 0.5 ± 0.05% iodophor for 5 min and (2) exposed to phosphate-buffered saline for 5 min. To gauge the level of biofilm, colony forming units (CFU), live/dead staining confocal microscopy and crystal violet staining were used. Meanwhile, the expression of *icaACDR* and *clfA,* which are related to virulence and adhesion, was examined in both the experimental and control groups.

**Results:**

A roughly three-log decrease in CFU/cm^2^ was seen in the viable plate count compared to the control group. Confocal imaging and crystal violet staining verified the CFU data. Moreover, the expression of *icaACDR* was reduced on three different orthopaedic implant materials, and the expression of *clfA* was also inhibited on titanium alloy coupons exposed to the iodophor.

**Conclusions:**

Our results indicated that exposure to an iodophor for 5 min could significantly eliminate biofilms. When *Staphylococcus aureus* that had adhered to these three materials, which were used for artificial joints, was treated with an iodophor for 5 min, the expression of *icaACDR* was significantly reduced. This provides strong evidence for clinically clearing periprosthetic joint infections without removing the artificial joints.

**Supplementary Information:**

The online version contains supplementary material available at 10.1186/s13018-023-04246-x.

## Introduction

Periprosthetic joint infection (PJI) is one of the most serious complications after artificial joint replacement (AJR) [1]. Over 98% of PJIs are caused by bacterial species, most of which are *Staphylococcus aureus* and coagulase-negative staphylococci, which account for 50% to 60% of all PJIs [2, 3]. Notably, a variety of factors, including the surface characteristics of the orthopaedic implant material, can affect bacterial adherence and facilitate infection [4]. *Staphylococcus aureus* (*S. aureus*) is the most frequent cause of infection based on implant biomaterials [5, 6].

A number of infections are biofilm-related [7, 8]. A biofilm is an aggregation of surface-associated microbial cells encased in an extracellular polymeric substance (EPS) matrix that provides mechanical stability and resistance to environmental hazards [9, 10]. Furthermore, biofilm bacteria are 100–1000 times less susceptible to antibiotics than planktonic bacteria [11]. It is very challenging to eradicate microbes or mechanically remove biofilms from solid surfaces after the biofilms have grown [12, 13].

Debridement, antibiotics, and implant retention (DAIR) are increasingly used for acute PJIs due to less invasion and lower cost compared to two-stage device exchange. On the other hand, DAIR appears to have a higher failure rate (16–57.4%), and *S. aureus* PJI appears to have a lower success rate than other organisms [3, 14–16].

Studies have shown that many different debridement techniques, such as iodine immersion, pulse lavage (PL), and even mechanical brushing, have been used in vitro to mechanically disrupt and remove the bacterial biofilm established on implant materials [17, 18]. Iodophors are well known as an antiseptic and exhibit extensive activity against various pathogens [19]. It has also been proven that iodophors have potency against fully developed bacterial biofilms in vitro and ex vivo [20, 21]. Combining an iodophor with vancomycin is superior in reducing viable *S. aureus* cells in immature biofilms grown on titanium surfaces without causing significant cytotoxicity to muscle tissue [22]. Iodophors were found to eliminate bacterial growth on the surface of contaminated polyethylene implants, while hydrogen peroxide failed in one case to completely eradicate growth [23]. Rough cobalt chromium molybdenum (CoCrMo) surfaces are prone to biofilms showing more proteins and polysaccharides, while the effect of iodophor treatment is still unknown [24]. No one has studied the effect of iodophor on biofilm elimination from titanium alloy (TA), cobalt chromium molybdenum (CoCrMo), and polyethylene, which are commonly used materials for artificial joints, and compared the differences in their efficacy.

The literature states that with regard to irrigant efficacy against biofilms, povidone-iodine treatment with the application of 20 ml for 1 min results in greater reductions in nascent MRSA biofilms compared to other solutions [25]. There is also literature that determined the minimum effective exposure time required to prevent the growth of *Staphylococcus aureus* povidone-iodine 0.35% (Betadine) after 90 s of treatment [26]. The iodophor instructions state that the time of action of skin disinfection at the surgical site is 2 min, the time of action at injection and puncture sites is 2 min, that for disinfection of the hands before surgery is 3 min, and that for disinfection of infected sites is 3 min. We designed a 24-h biofilm infection model and carefully synthesized all the references to determine the iodophor duration of action to be 5 min.

Many studies have shown that the inhibition of biofilms by iodophors was associated with a decrease in transcription of the *icaADBC* operon, which in turn correlated with the activation of the *icaR* transcription inhibitor in *S. aureus* [27–30]. Bacterial adherence to the target cell is the primary stage of infection. It is determined by *fnbA* and *fnbB* (encoding fibronectin-binding proteins A and B), *fib* (encoding fibrinogen-binding proteins), *cna* (encoding collagen-binding protein), *clfA* and *clfB* (encoding clumping Factors A and B) and *eno* (encoding laminin-binding protein) [31]. Adherence or attachment ability and biofilm production are important for enhancing virulence factors among isolates of *S. aureus* [32, 33]. These studies have shown that iodophors can affect the expression of the *icaADBC* operon and that the virulence and adhesion of *S. aureus* may also be affected by *icaR*. *clfA*, *clfB*, *fnbA*, *fnbB,* etc.

Titanium alloy (TA), cobalt chromium molybdenum (CoCrMo), and polyethylene are commonly used materials for artificial joints. Only one study has investigated the effect of iodophor treatment on biofilm elimination from TA, CoCrMo, and polyethylene materials, but none have investigated the expression of adhesion and virulence-related genes of *S. aureus* after iodophor treatment. Therefore, we assume that an iodophor acts on the surface of the prosthesis to remove the biofilm and has an effect on the expression of adhesion- and virulence-related genes. In addition, there is the question of whether the effect differs for different implant materials. Therefore, we extended the study to biofilms grown on TA, CoCrMo, and polyethylene coupons and compared the efficacy of eradicating *S. aureus* biofilms by using an iodophor. Next, we analysed the expression of *clfA, icaA, icaB, icaD*, and *icaR*, which are related to the adhesion and virulence of *S. aureus*. If the treatment can reduce the expression of biofilm adhesion and virulence genes, we will be able to study the molecular mechanism of biofilm elimination, which may lead to therapeutic targets for biofilms. If it can actually remove the biofilm on the surface of the prosthesis, this will significantly improve the success rate of DAIR, which is unquestionably good news for PJI patients.

## Materials and methods

### Bacterial strain

This study used *S. aureus* ATCC 25923, provided by the Department of Clinical Laboratory, Jiaxing First Hospital. The strain was grown on tryptic soy agar (TSA; Fushenbio, Shanghai, China) at 37 °C and 5% CO_2_. Then, representative colonies were picked and suspended in trypticase soy broth (TSB; Fushenbio, Shanghai, China), growing at 37 ℃ overnight with agitation (200 rpm). Bacteria were harvested and resuspended in TSB, adjusted to the turbidity equivalent to 1 McFarland and diluted 1:300, achieving a final cell concentration of approximately 1 × 10^6^ CFU/mL.

### Biofilm formation on orthopaedic implants

Titanium alloy (TA), cobalt chromium molybdenum (CoCrMo), and polyethylene materials were made into 10 × 10 × 1 mm smooth coupons (AK Medical Ltd, Beijing, China), washed with an ultrasonic water washing instrument for 30 min before use, and autoclaved after drying. The coupons were placed in 24-well clear bottom microtiter plates (Corning Inc., Corning, NY). Subsequently, 1 mL of bacterial suspension was added to each well and incubated for 24 h at 37 °C and 5% CO_2_.

### Iodophor exposure

After 24 h, the coupons were removed from the bacterial suspension, followed by either (1) exposure to 0.5 ± 0.05% iodophor (Health Essence, Beijing, China) for 5 min or (2) exposure to phosphate buffered saline (PBS; Solarbio, Beijing, China) for 5 min. Experiments were performed in triplicate.

### Viable cell count

Each coupon was rinsed with sterile PBS after being exposed to the treatment arms. The rinsed coupon was placed in a 15-mL tube containing 10 mL of PBS. By applying a 35 kHz sonication frequency to 10 mL of PBS for 15 min, the biofilm was eliminated in 10 mL of PBS. A 10-s vortex interval was added between each of the three times sonication was performed. A total of 10 serial dilutions were made and plated onto TSA, which was then incubated for 24 h at 37 °C with 5% CO_2_. The number of CFUs, represented as CFU/cm^2^ was then determined.

### Biofilm assays

The wells were treated for 5 min with iodophor and PBS, and after that, all of the bacteria in the basal state were eradicated by giving the wells two rounds of PBS rinsing. To stain the biofilm, 1 mL of 0.1% crystal violet staining solution (Beyotime, Shanghai, China) was applied to each well after the plates were dried at 37 °C. At room temperature, the plates were incubated for 15 min. After the stain was removed, the plates were flushed three times with PBS and then dried at 37 °C. One millilitre of 95% ethanol was used to dissolve the biofilm. Using a microplate reader (ELX800, Bio-Tek, USA), the absorbance was measured at a wavelength of *λ* = 570 nm after 15 min of incubation.

### Confocal laser scanning microscopy (CLSM)

Confocal laser scanning microscopy (CLSM, LSM800, Zeiss, Germany) was used in the control group and treatment group to image the bacterial biofilms and confirm the CFU data. The bacterial biofilms were observed using a SYTO-9/PI Live/Dead Bacterial Double Stain Kit (Fushenbio, Shanghai, China) following the manufacturer’s instructions. The Live-Dead kit contains SYTO-9, which stains viable bacterial DNA green, and dead cells appear red when propidium iodide (PI) enters compromised bacterial cell membranes. After exposure to the treatments, the coupons were lightly dipped in sterile water three times to remove nonfirmly attached bacteria and debris. Then, after staining for 15 min at room temperature in the dark, the biofilms were rinsed with PBS to remove the extracellular dyes and observed with CLSM.

### Determination of gene expression by quantitative real-time PCR (q-PCR)

#### Ribonucleic acid (RNA) isolation

The bacterial culture was centrifuged (5 min, 6000 RCF) after 24 h of incubation. Total RNA was subsequently isolated using a Bacteria Total RNA Isolation Kit (Sangon Biotech, Shanghai, China) according to the manufacturer’s protocol following a 5–10 min pretreatment of the cells with 50 mg of lysostaphin.

(Sangon Biotech, Shanghai, China) in 100 mL of 50 mM EDTA. Purified RNA was eluted with DNase/RNase-Free Water (Beyotime, Shanghai, China), and the integrity of the RNA was confirmed by 1% agarose gel electrophoresis. RNA quantity and purity were determined by a Microvolume UV‒Vis Spectrophotometer (NanoDrop One, Thermo Fisher Scientific, USA).

#### Reverse transcription

The reverse transcription reaction was performed using 5 × PrimeScript™ RT Master Mix (Perfect Real Time) (Takara Biomedical Technology, Beijing, China).

The reaction mixture (20 μL) contained is given in Table 1.

**Table 1:** Components for reverse transcription

5 × PrimeScript™ RT Master Mix (Perfect Real Time)	4 μL
DNase/RNase-free water	14 μL
Template RNA (approximately 5–500 ng per 1 μL)	2 μL
Total volume	20 μL

After gentle mixing, reverse transcription was carried out under the following conditions: 37 °C for 15 min and 85 °C for 5 s.

#### Q-PCR

q-PCR was performed to detect the five genes related to biofilm formation capacity*, clfA, icaA, icaB, icaD, and icaR,* and the *16S* reference gene (Table 2).

**Table 2:** Gene sequences for real-time PCR

Gene	Nucleotide sequence (5′–3′)
*clfA*	F: CAAGTAGCGTTAGTGCTGC
	R: TGATTGAGTTGTTGCCG
*icaA*	F: CTATTTCGGGTGTCTTCACTC
	R: GGCAAGCGGTTCATACTTA
*icaB*	F: TTGCCTGTAAGCACACTGGATGGTC
	R: TACACGGTGATAATTTAATGCCAGAGC
*icaD*	F: ATGGACAAGTCCAGACAGAGGAAAA
	R: GTCACTCATCGTAACTGCTTCAACG
*icaR*	F: TCAGAGAAGGGGTATGACGGTACAA
	R: TCCTCAGGCGTATTAGATAATTGAACG
*16S*	F: CGTGCTACAATGGACAATACAAA
	R: ATCTACGATTACTAGCGATTCCA

For this purpose, PowerUp™ SYBR™ Green Master Mix (Thermo Fisher Scientific, USA) was used, following the manufacturer’s recommended protocol. The reaction mixture (20 μL) contained is given in Table 3.

**Table 3:** Components for real-time PCR

PowerUp™ SYBR™ green master mix	10 μL
DNase/RNase-free water	6 μL
Forwards primers	1 μL
Reverse primers	1 μL
Template cDNA (approximately 5–100 ng per 1 μL)	2 μL
Total volume	20 μL

Run the program as follows (Tables 4, 5).

**Table 4:** Standard cycling mode for real-time PCR

Steg	Temperature (°C)	Duration	Cycles
UDG activation	50	2 min	Hold
Dual-Lock™ DNA polymerase	95	2 min	Hold
Denature	95	15 s	40
Anneal/extend	60	1 min

**Table 5:** Melt curve stage procedure for real-time PCR

Step	Ramp rate (°C)	Temperature	Time
1	95	15 s	1.6 °C/s
2	60	1 min	1.6 °C/s
3	95	15 s	0.15 °C/s

### Statistical analysis

GraphPad Prism 5 software (GraphPad Prism Software, Inc.; San Diego, CA, USA) was used for statistical analysis. A two-tailed, unpaired Student's t test with equal variance was used. If *p* < 0.01, statistical significance was determined.

## Results

### Viable cell count

**Fig. 1:**
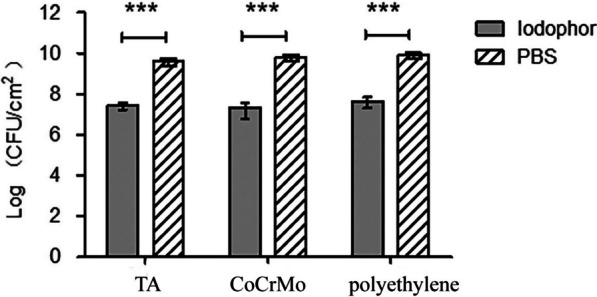
The biofilm cell density was measured after treatment, revealing a decrease in biofilm mass. *TA* titanium alloy, *CoCrMo* cobalt chromium molybdenum. *** represent *P* ≤ 0.001, and there is a statistically significant difference between the two sets of data when *P* ≤ 0.001

The number of CFUs on the coupons was used to quantify the bacteria. After 24 h, the viable cell count in the control group had grown to approximately 10^10^ CFU/cm^2^ (Fig. 1). The treatment group showed an approximate three-log reduction in CFU/cm^2^ compared to the control group (*p* < 0.001). However, there was not much difference between the CoCrMo coupons and TA coupons in the reduction of CFUs, and the polyethylene showed less reduction than the two orthopaedic implant materials.

### Determination of antibiofilm activity

**Fig. 2:**
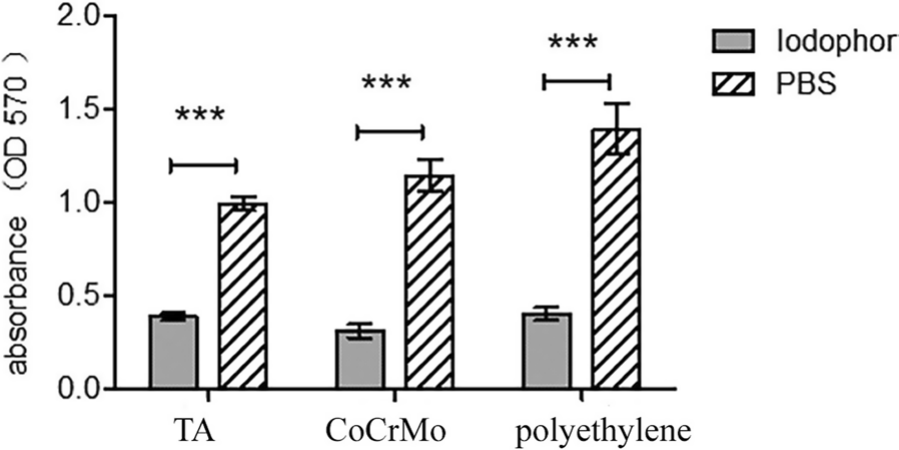
The biofilm absorbance was measured by a microplate reader, indicating a decrease in the amount of biofilm. *TA* titanium alloy, *CoCrMo* cobalt chromium molybdenum. *** represents *P* ≤ 0.001, and there is a statistically significant difference between the two sets of data when *P* ≤ 0.001

Biofilms formed by *Staphylococcus aureus* on the surface of different orthopaedic implant materials were quantified using crystal violet staining. The absorbance count represented the amount of biofilm formed. The amount of *Staphylococcus aureus* biofilm on the polyethylene surface was the largest, followed by that on CoCrMo and TA. After treatment, the absorbance of the biofilms was significantly reduced compared to that of the control group (*p* < 0.001). CoCrMo had the lowest absorbance values, while the other two materials had similar absorbance values. Polyethylene had the largest reduction in absorbance, followed by CoCrMo and TA (Additional file 1) (Fig. 2).

### Live/dead backlight staining by confocal microscopy

**Fig. 3:**
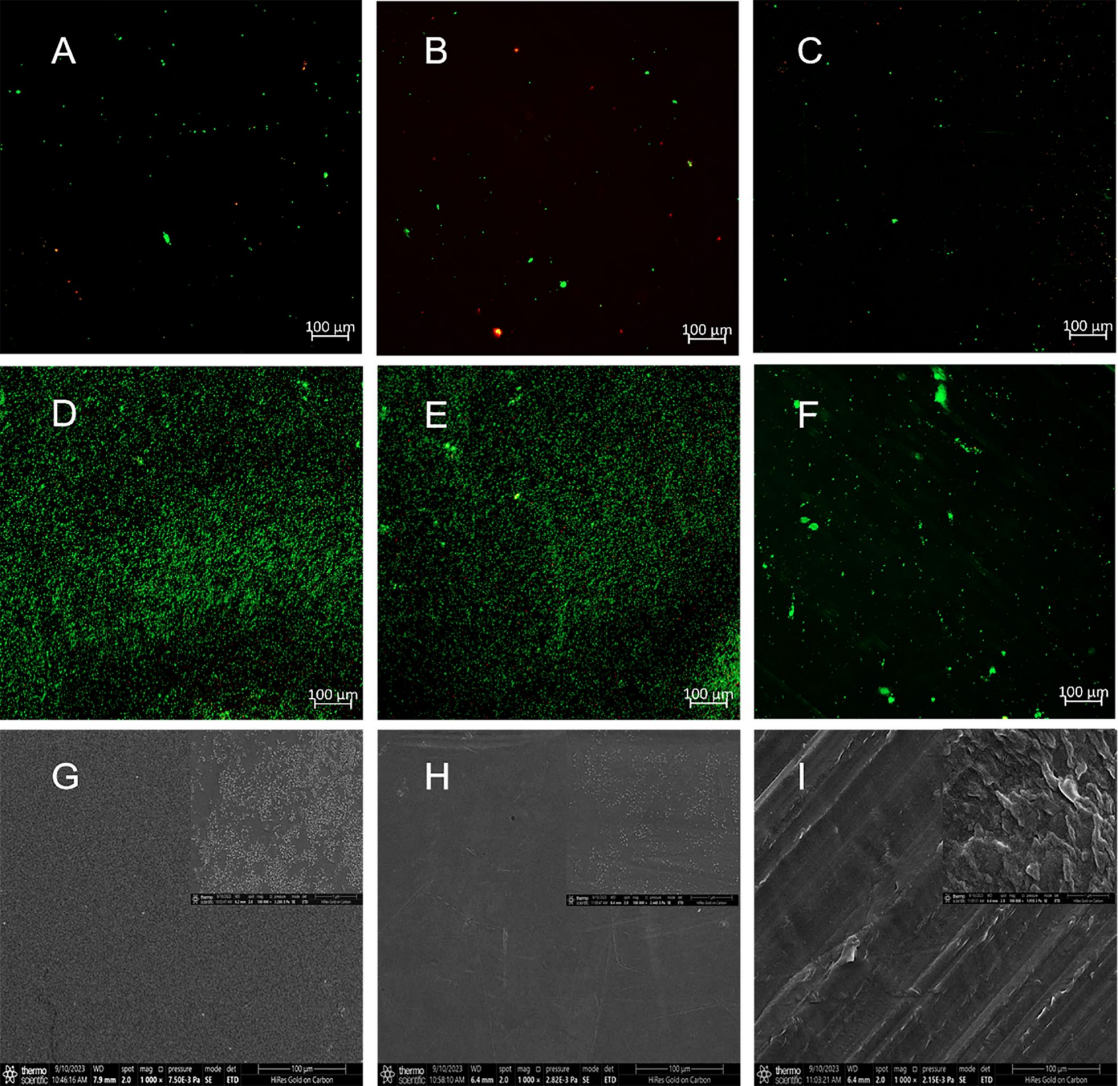
Confocal laser scanning microscopic images showing biofilm formation. (**A**: cobalt chromium molybdenum (CoCrMo) coupons exposed to the iodophor for 5 min; **B**: titanium alloy (TA) coupons exposed to the iodophor for 5 min; **C**: polyethylene coupons exposed to the iodophor for 5 min; **D**: cobalt chromium molybdenum (CoCrMo) coupons exposed to PBS for 5 min; **E**: titanium alloy (TA) coupons exposed to PBS for 5 min; **F**: polyethylene coupons exposed to PBS for 5 min.) Live cells are stained green, and dead cells are stained red. The scale bar represents 100 μm magnification. **G-I**: Scanning electron microscopy (SEM, FEI Inspect F50, ThermoFisher, USA) images to describe the surface characterization of materials. (**G**: cobalt chromium molybdenum (CoCrMo) coupons; **H**: titanium alloy (TA) coupons; **I**: polyethylene coupons.) Scale bars represent 100 μm and 1 μm magnification respectively

Confocal microscopy was a useful method for measuring the level of biofilm debridement after exposure to the iodophor for 5 min or PBS for 5 min (Fig. 3). After exposure to the iodophor for 5 min, the luminescence of the artificial prosthesis materials was reduced. This means that the biofilm cell density and viability had been reduced. Polyethylene showed less green staining due to light transmission than CoCrMo coupons and TA coupons, either for 5 min of iodophor treatment or PBS (Additional file 2).

### Determination of gene expression by real-time PCR

**Fig. 4:**
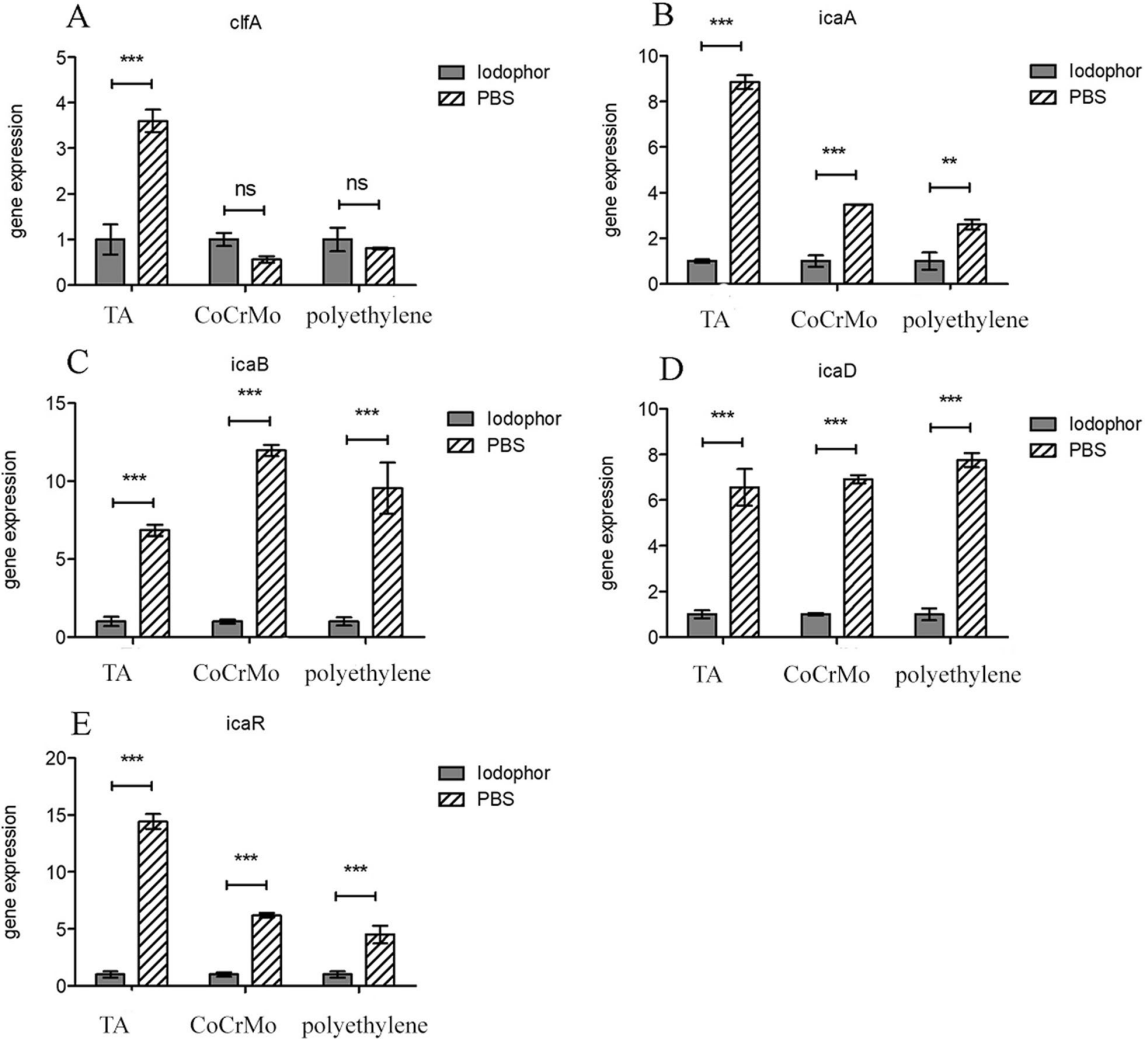
Comparative measurement of *clfA*, *icaA*, *icaB*, *icaD* and *icaR* transcription in the *S. aureus* ATCC 25923 strain. *TA* titanium alloy, *CoCrMo* cobalt chromium molybdenum. Ns, ** and *** represent *P* > 0.05, *P* ≤ 0.01, *P* ≤ 0.001, and there is a statistically significant difference between the two sets of data when *P* ≤ 0.01

Using real-time PCR, the levels of relative gene expression were measured to determine whether the reduced ability to build biofilms in the presence of the iodophor was associated with altered expression of the *icaABDR* and *clfA* loci. Consistent with the reduction in biofilm, *icaABDR* expression is reduced on three artificial joint materials and *clfA* expression is also inhibited on titanium alloy coupons exposed to the iodophor (Additional file 1). The results are statistically significant (*p* ≤ 0.01) (Fig. 4).

## Discussion

The development of biofilms and bacterial proliferation causes an implant infection that is resistant to treatment [34]. There is an urgent need for an efficient method to remove bacterial biofilms from implants due to the significant risks of infection that could develop during the perioperative period [35]. The primary method of treating acute periprosthetic infection is surgical debridement in conjunction with certain antibiotics. For the clinical diagnosis and management of periprosthetic infections, research on the antibacterial properties of iodophors on various orthopaedic implant materials is beneficial.

In vitro, the scavenging effect of some disinfectants on biofilms has been studied. A high concentration of farnesol (30 mM) shows antimicrobial properties against bacterial biofilms [36, 37]. Hypochlorous acid, a powerful disinfectant, can break bacterial biofilms and may be useful in the treatment of orthopaedic fixative infections [38]. Hydrogen peroxide also has the same effect [39]. These results indicate that disinfectants can remove biofilms. Iodophors are also effective in removing biofilms, which supports our research [26, 27, 40].

This is the first study of the effect of iodophors on biofilm clearance from artificial joint materials. Our study revealed that the number of *S. aureus* organisms and the number of biofilms on the surface of TA, CoCrMo, and polyethylene coupons decreased significantly after 5 min of iodophor treatment, and the number of plate counts decreased from 10^10^ to 10^7^. Several studies have shown that iodine-supported titanium implants can inhibit biofilm formation [41, 42]. Polyethylene joint prostheses are prone to infection and wear [43]. Rough cobalt chromium molybdenum (CoCrMo) surfaces are prone to biofilms showing more proteins and polysaccharides, while the effect of iodophor treatment is still unknown [24]. Perhaps the addition of iodine coatings to the other two materials will make the biofilm removal effective.

Grossman quantified *S. aureus* biofilm formation by crystal violet and confocal microscopy, which is a classic and standardized approach [44]. We used the same methodology for our research. After iodophor treatment, the number of biofilms in CoCrMo coupons was the lowest, followed by the TA coupons and the polyethylene coupons. Before treatment, the TA coupons had the lowest number of biofilms, followed by the CoCrMo coupons, and the polyethylene coupons had the highest. Regardless of whether the polyethylene coupon was treated, its biofilm adherence was greatest.

The results of live/dead backlight staining by confocal microscopy revealed a large number of viable bacteria (green light) on the surface of the three orthopaedic implant materials in the PBS treatment group. Comparatively, the number of viable bacteria on the surface of the three materials in the iodophor treatment group was significantly reduced to only a small number, and there were few dead bacteria (red light). Moreover, polyethylene coupons showed less fluorescence due to light transmission than the other two coupons.

PI was shown to significantly inhibit the formation of bacterial biofilms and reduce the expression of the *hla*, *ebps*, *eno*, *fib*, *icaA*, and *icaD* genes [40]. The results for *icaA* and *icaD* are consistent with ours. Our data revealed that in addition to its known antibacterial properties, iodophors can also inhibit *S. aureus* biofilm development at least in part by repressing the transcription of *icaABDR,* and the transcription of *clfA* was inhibited on titanium alloy coupons. Oduwole and Barakat's articles also confirmed this finding [30, 39]. The expression of the 5 genes had different inhibitory effects on different materials.

However, our study was limited in some ways. First, we did not evaluate modifications to the implant surface, such as surface roughness, which has a significant impact on implant longevity [45, 46]. And surface roughness also affects the total surface area which in turn can influence biofilms formation. Second, it may not apply to other types of bacteria, such as methicillin-resistant *Staphylococcus aureus* (MRSA), as we used only one strain experiment model. Third, the optimal action time and concentration of iodophors need to be further experimentally explored. More rigorous animal experiments and large-sample, multicentre, randomized controlled clinical trials are also needed, which will be carried out in the future.

In addition, it should be noted that the PCR technique may be insufficient to determine the genetic basis of biofilm production because the presence or absence of a gene does not directly indicate that the encoded protein plays a role in the ability of the staphylococci to form a biofilm in the first place. Therefore, future research should also focus on the use of advanced molecular biology techniques to gain a better understanding of the genetic basis of adhesion and virulence capacity in the *Staphylococcus* genus.

## Conclusion

This is the first study to explore the inhibition of *S. aureus* biofilms and the expression of genes related to adhesion and virulence when artificial joint replacement materials (titanium alloy, cobalt chromium molybdenum, polyethylene) are exposed to the iodophor. An in vitro study showed that artificial joint materials with adherent* S. aureus* showed significant elimination of biofilms when exposed to an iodophor for 5 min, and the expression of *icaACDR* was significantly reduced. Moreover, iodophor-treated titanium alloys reduced the expression of *clfA*. This provides strong evidence for using iodophors clinically to treat periprosthetic joint infections without removing the artificial joints. More experimental and in vivo studies are needed to support this hypothesis in the future.

### Supplementary Information

**Additional file 1.** Original experimental data.

**Additional file 2.** Original SEM photo data.

## Data Availability

The analysed dataset from this investigation is available from the corresponding author upon reasonable request.

## Abbreviations

PJI: Periprosthetic joint infection
TA: Titanium alloy
CoCrMo: Cobalt chromium molybdenum
AJR: Artificial joint replacement
EPS: Extracellular polymeric substance
DAIR: Debridement, antibiotics, and implant retention
PL: Pulse lavage
TSA: Tryptic soy agar
TSB: Trypticase soy broth
CFU: Colony forming unit
PBS: Phosphate-buffered saline
CLSM: Confocal laser scanning microscopy
PI: Propidium iodide
RNA: Ribonucleic acid
q-PCR: Quantitative real-time polymerase chain reaction
MRSA: Methicillin-resistant *Staphylococcus aureus*
