# Supplementary figures and images for: The effect and mechanism of iodophors on the adhesion and virulence of Staphylococcus aureus biofilms attached to artificial joint materials
Source: J Orthop Surg Res. 2023 Oct 5;18:756. doi: 10.1186/s13018-023-04246-x (PMC10557172; doi:10.1186/s13018-023-04246-x)

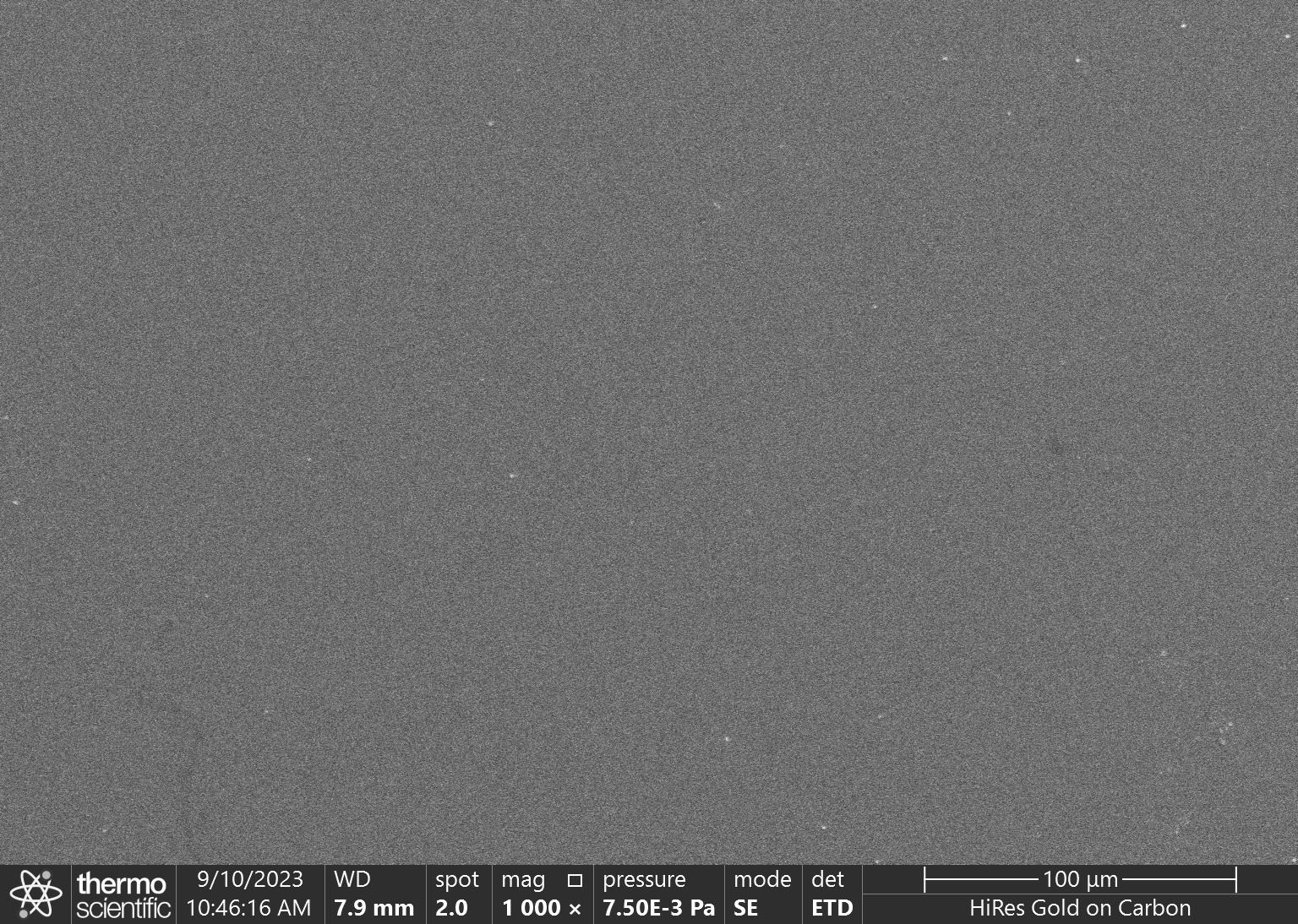

Supplement: Supplementary file 1 — Additional file 1. Original experimental data. [file 13018_2023_4246_MOESM1_ESM.zip › SEM/CoCrMo (1).tif]

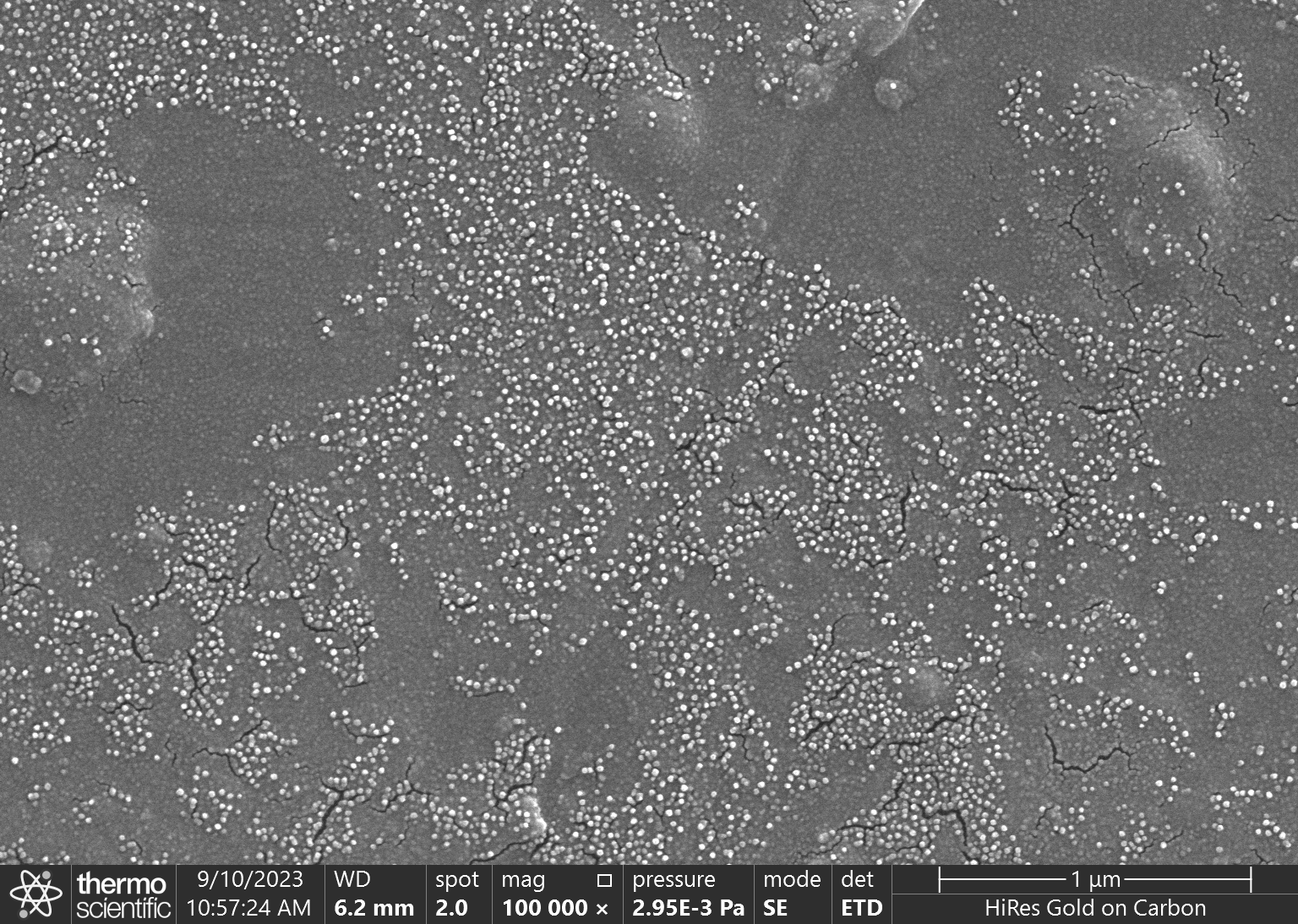

Supplement: Supplementary file 1 — Additional file 1. Original experimental data. [file 13018_2023_4246_MOESM1_ESM.zip › SEM/CoCrMo (10).tif]

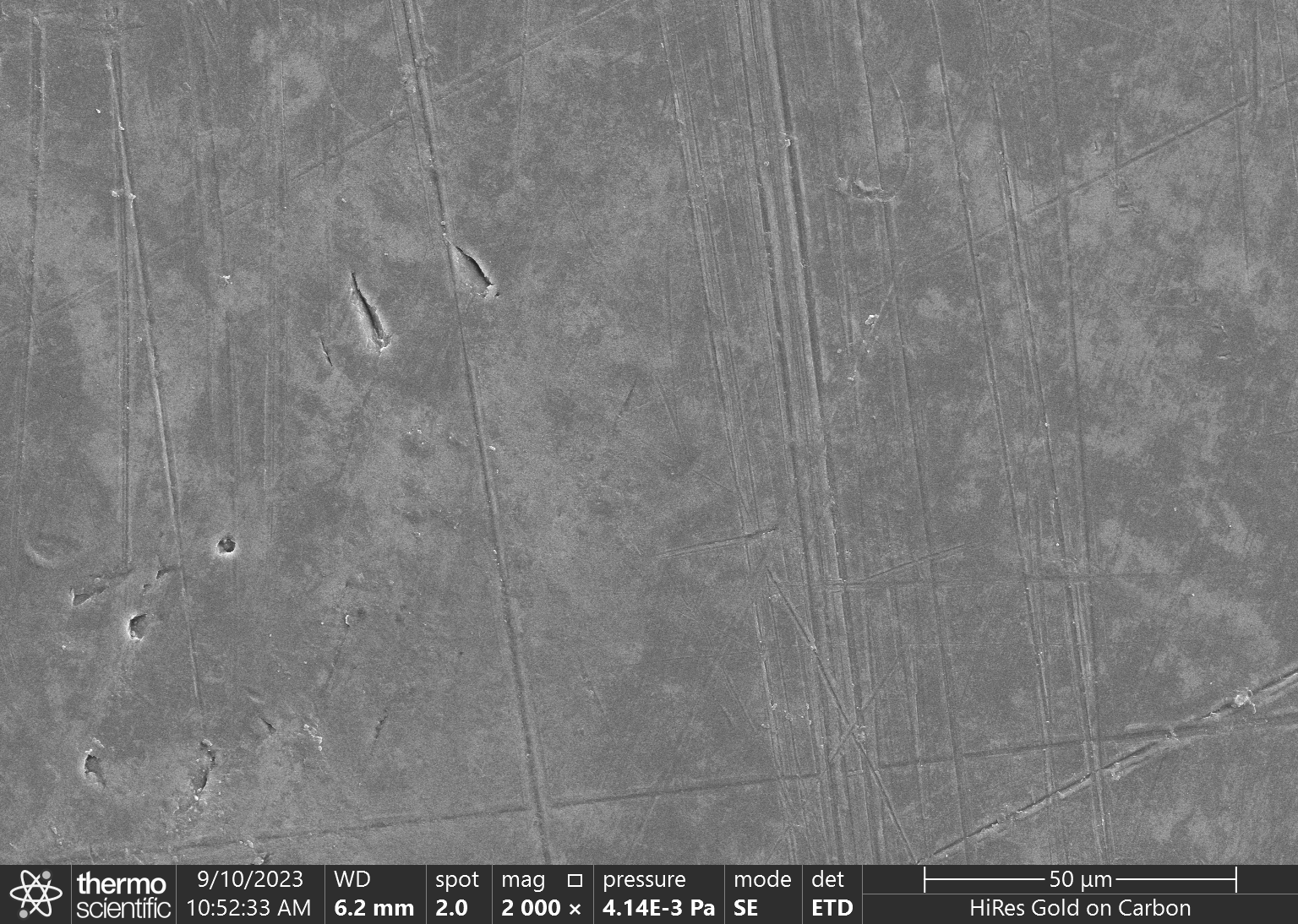

Supplement: Supplementary file 1 — Additional file 1. Original experimental data. [file 13018_2023_4246_MOESM1_ESM.zip › SEM/CoCrMo (2).tif]

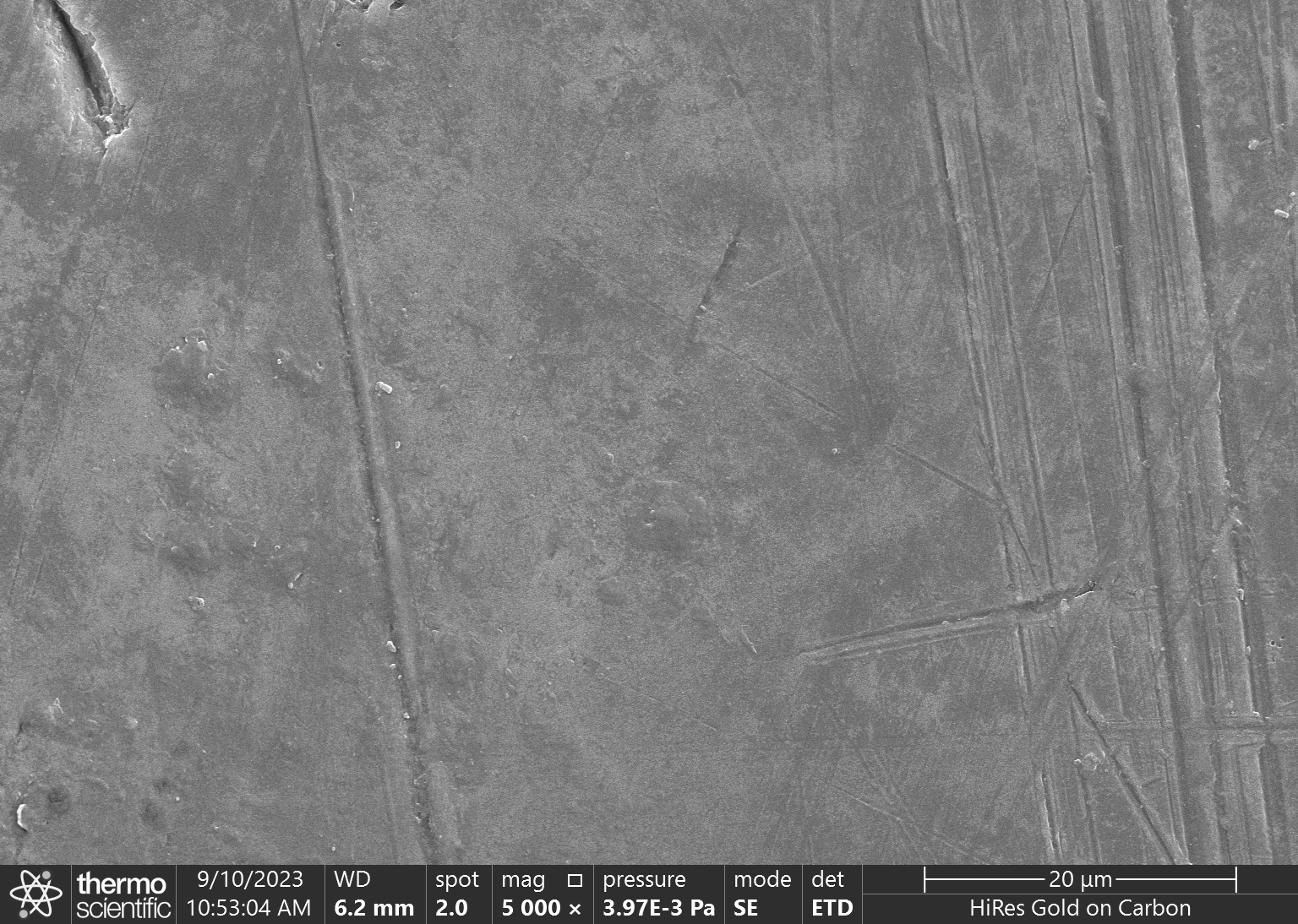

Supplement: Supplementary file 1 — Additional file 1. Original experimental data. [file 13018_2023_4246_MOESM1_ESM.zip › SEM/CoCrMo (3).tif]

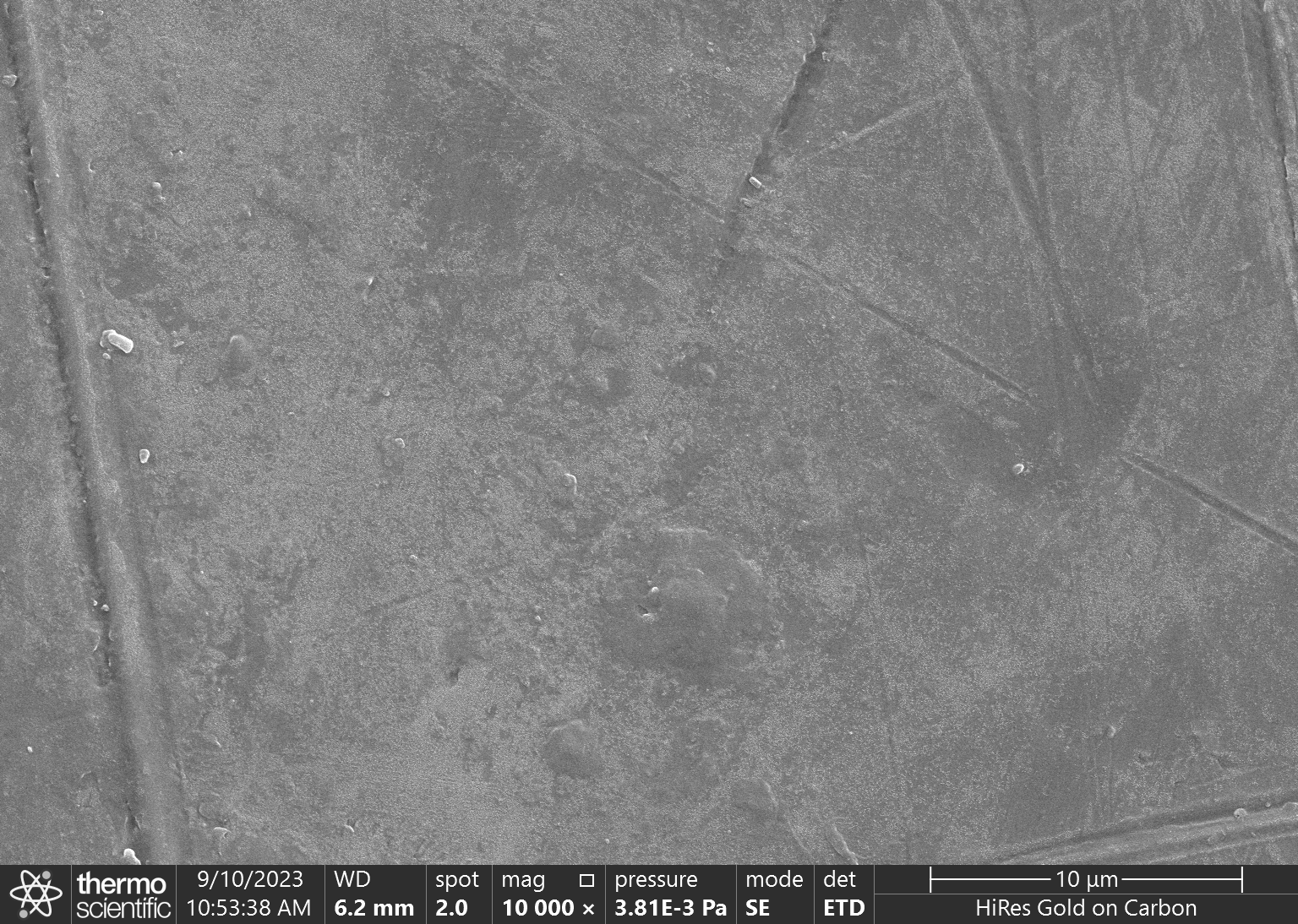

Supplement: Supplementary file 1 — Additional file 1. Original experimental data. [file 13018_2023_4246_MOESM1_ESM.zip › SEM/CoCrMo (4).tif]

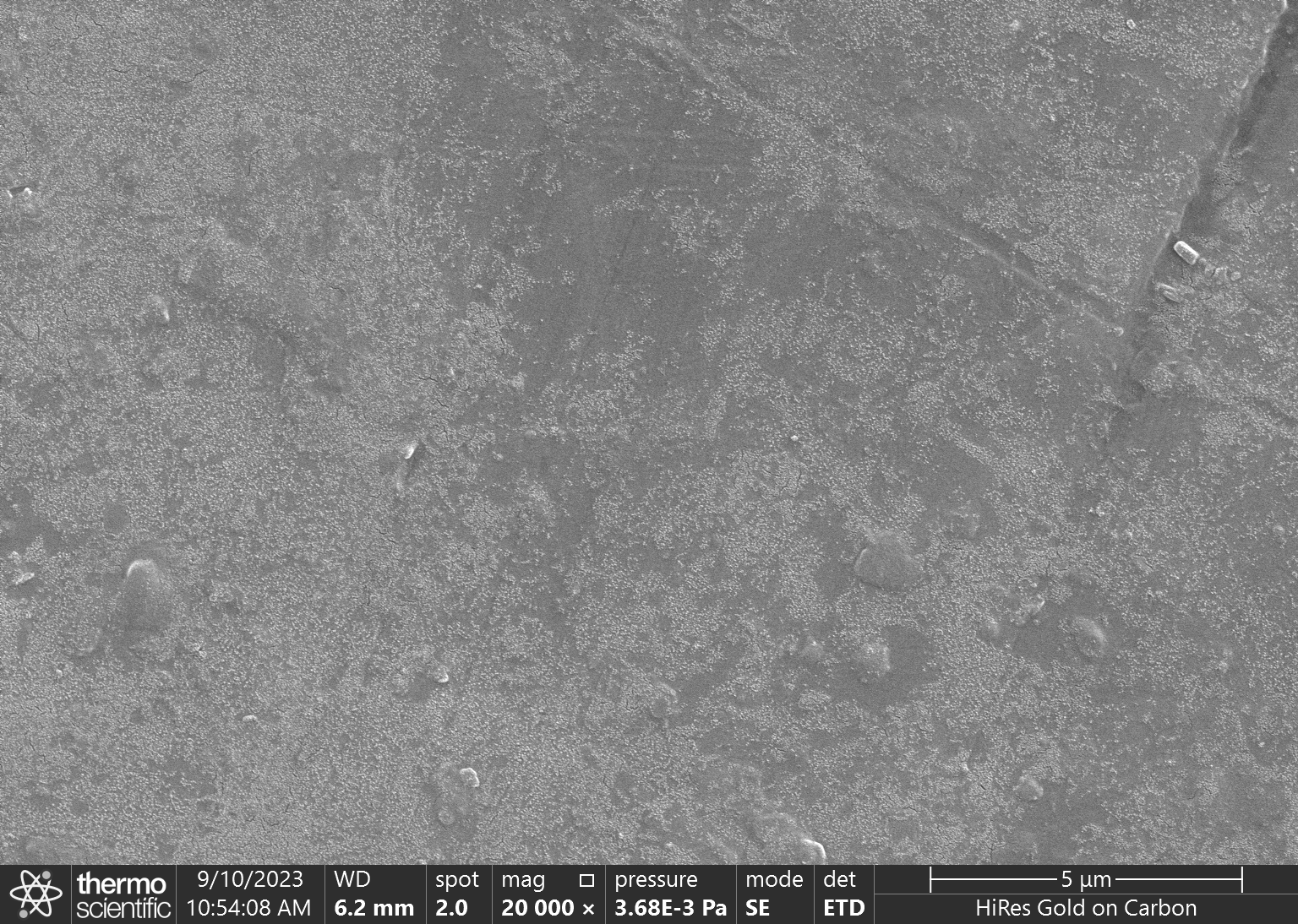

Supplement: Supplementary file 1 — Additional file 1. Original experimental data. [file 13018_2023_4246_MOESM1_ESM.zip › SEM/CoCrMo (5).tif]

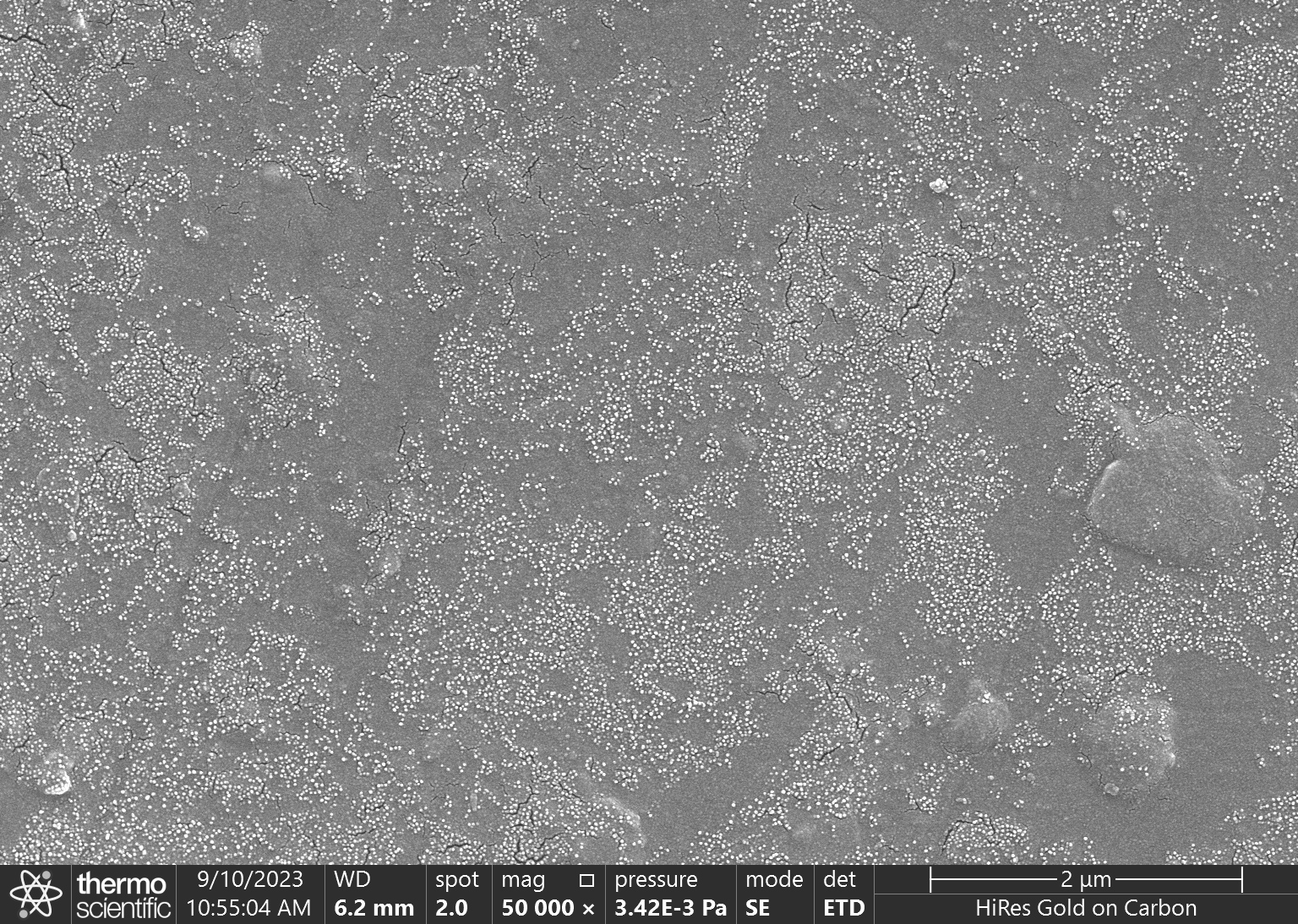

Supplement: Supplementary file 1 — Additional file 1. Original experimental data. [file 13018_2023_4246_MOESM1_ESM.zip › SEM/CoCrMo (6).tif]

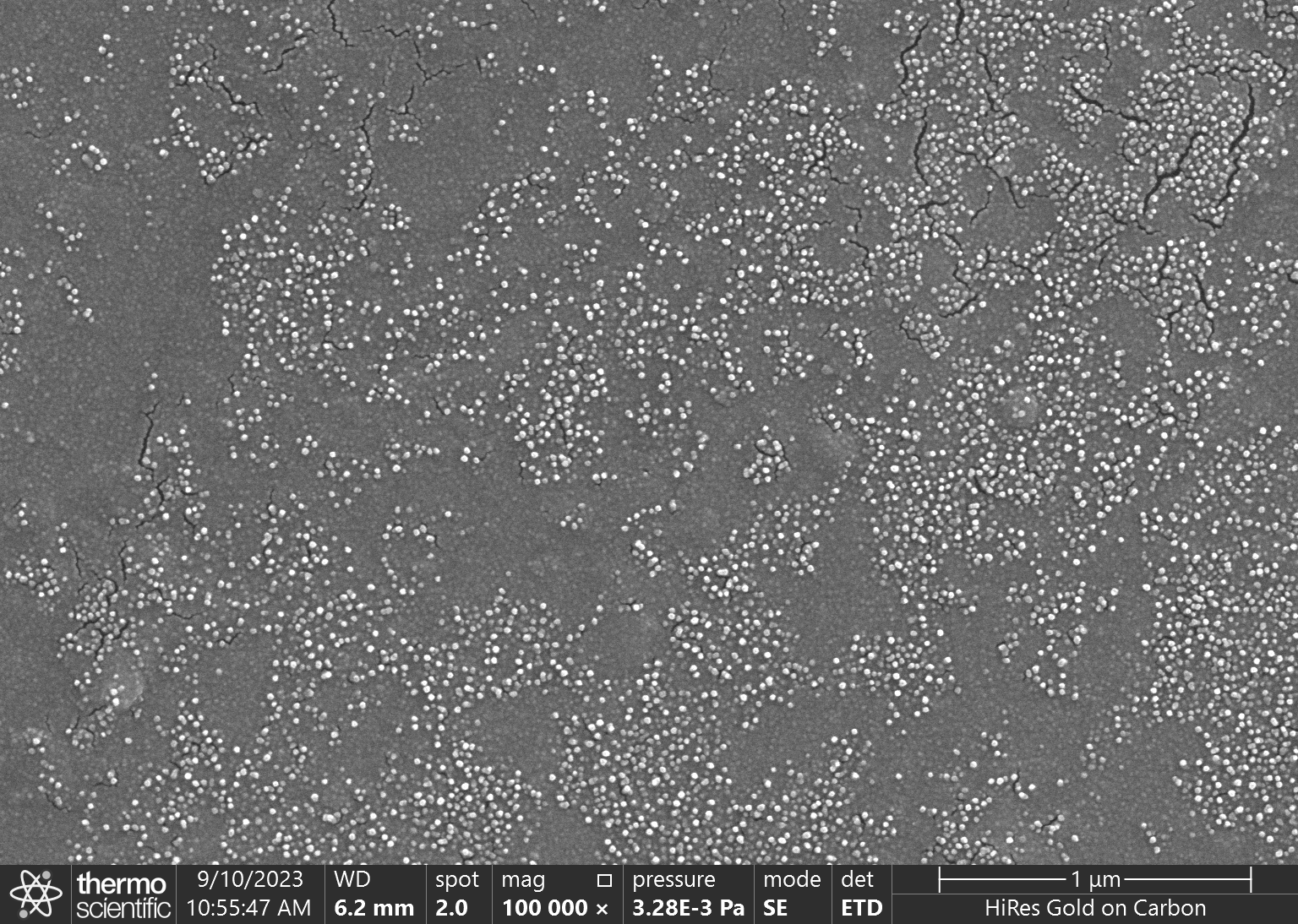

Supplement: Supplementary file 1 — Additional file 1. Original experimental data. [file 13018_2023_4246_MOESM1_ESM.zip › SEM/CoCrMo (7).tif]

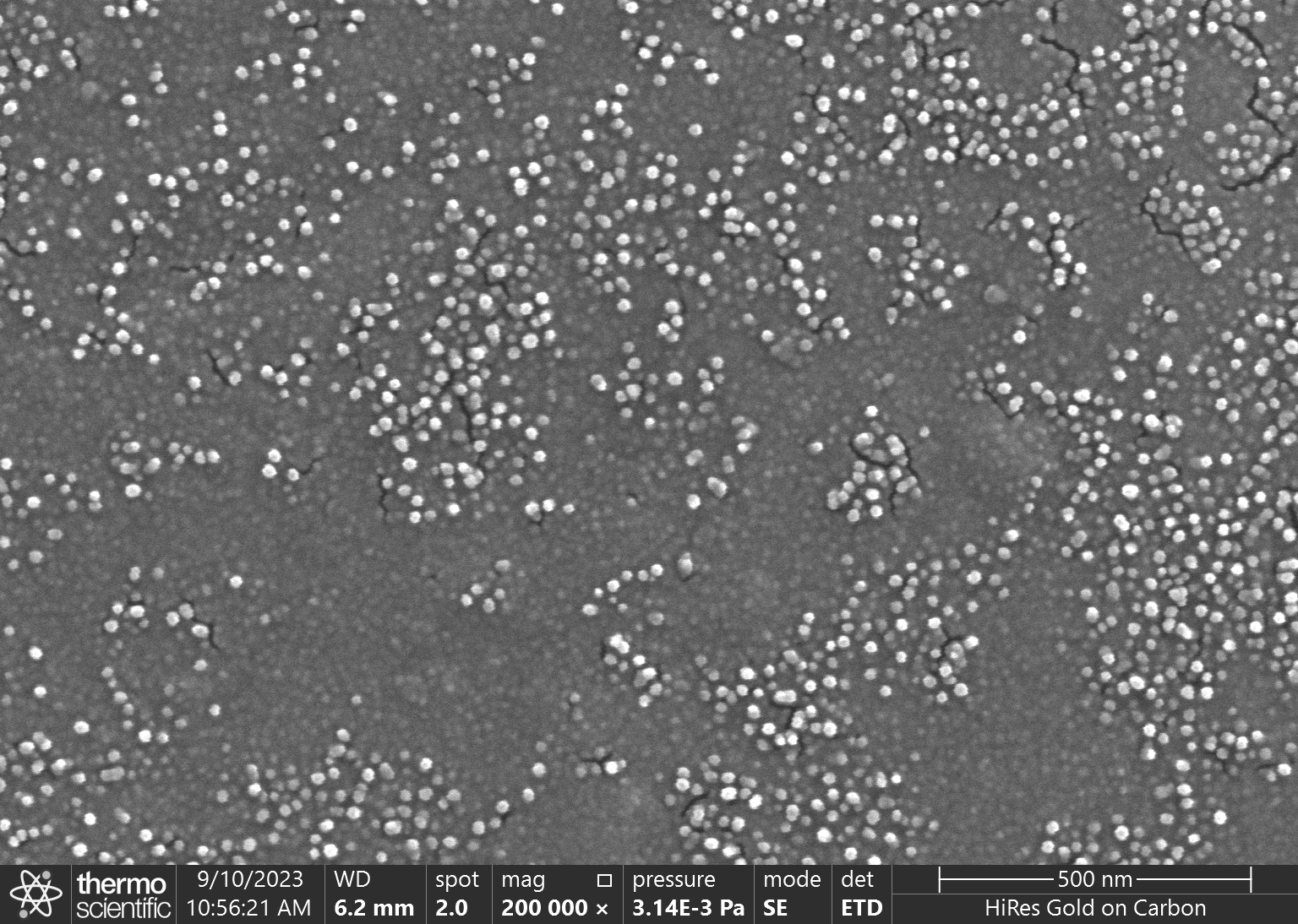

Supplement: Supplementary file 1 — Additional file 1. Original experimental data. [file 13018_2023_4246_MOESM1_ESM.zip › SEM/CoCrMo (8).tif]

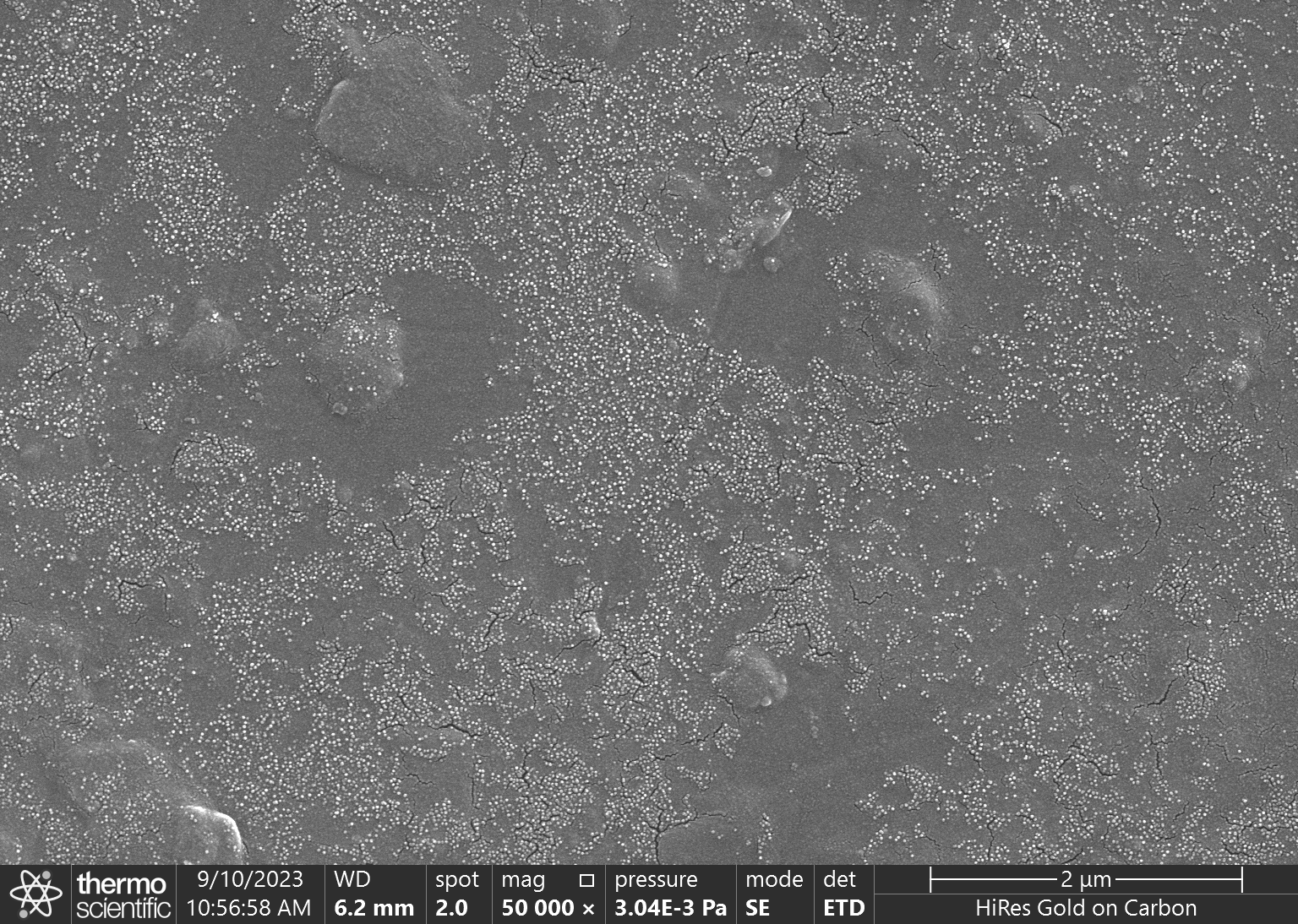

Supplement: Supplementary file 1 — Additional file 1. Original experimental data. [file 13018_2023_4246_MOESM1_ESM.zip › SEM/CoCrMo (9).tif]

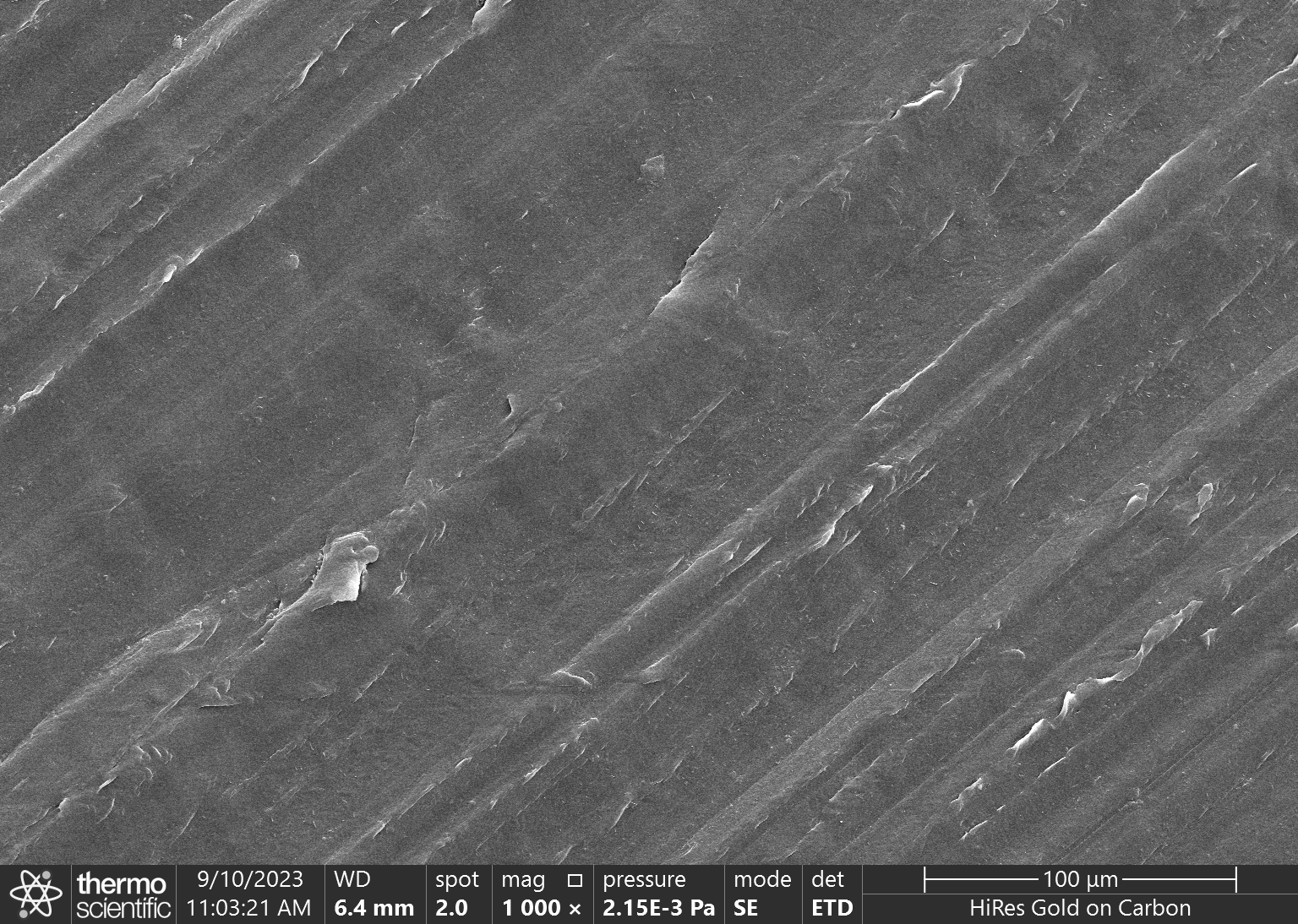

Supplement: Supplementary file 1 — Additional file 1. Original experimental data. [file 13018_2023_4246_MOESM1_ESM.zip › SEM/polyethylene (1).tif]

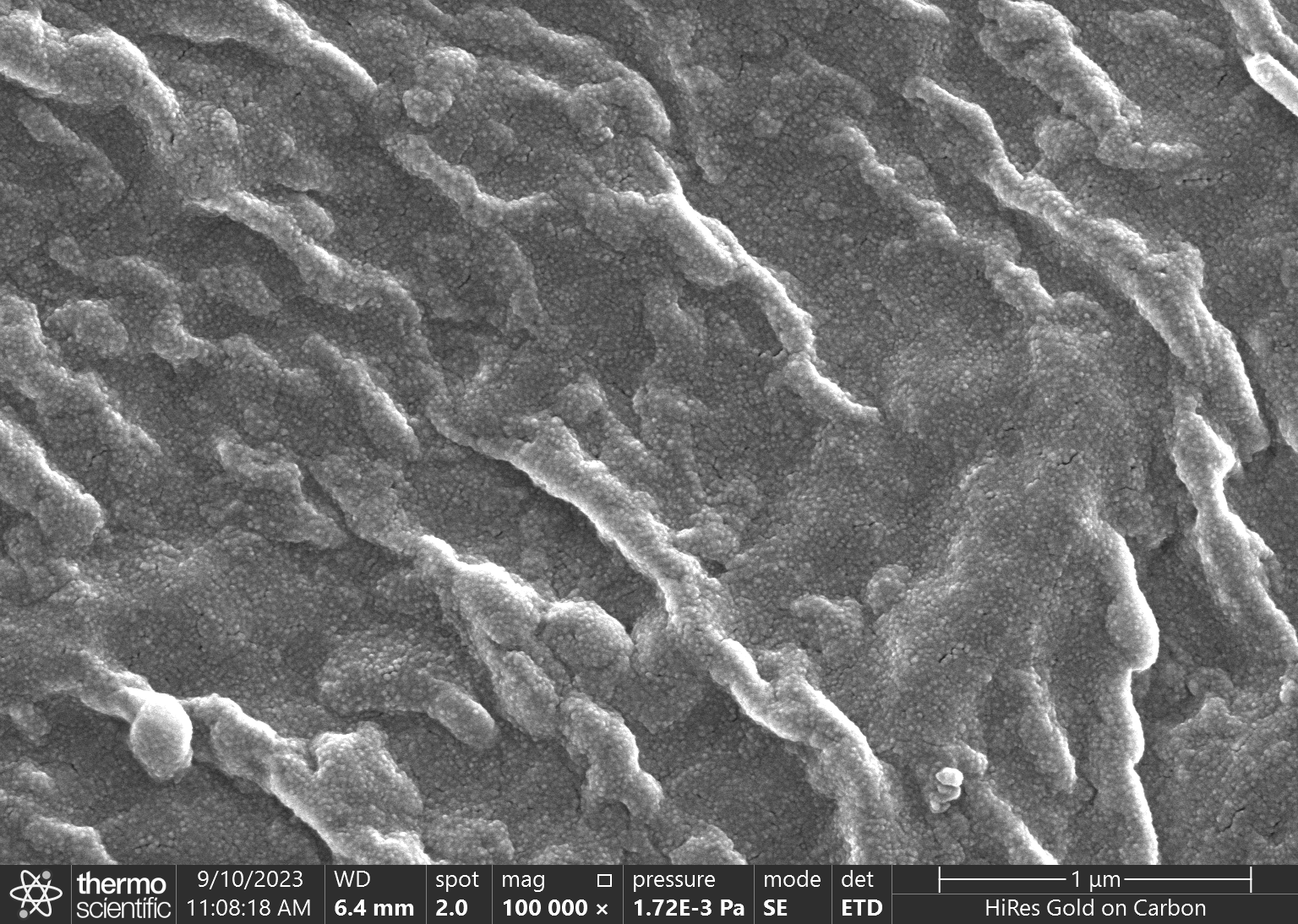

Supplement: Supplementary file 1 — Additional file 1. Original experimental data. [file 13018_2023_4246_MOESM1_ESM.zip › SEM/polyethylene (10).tif]

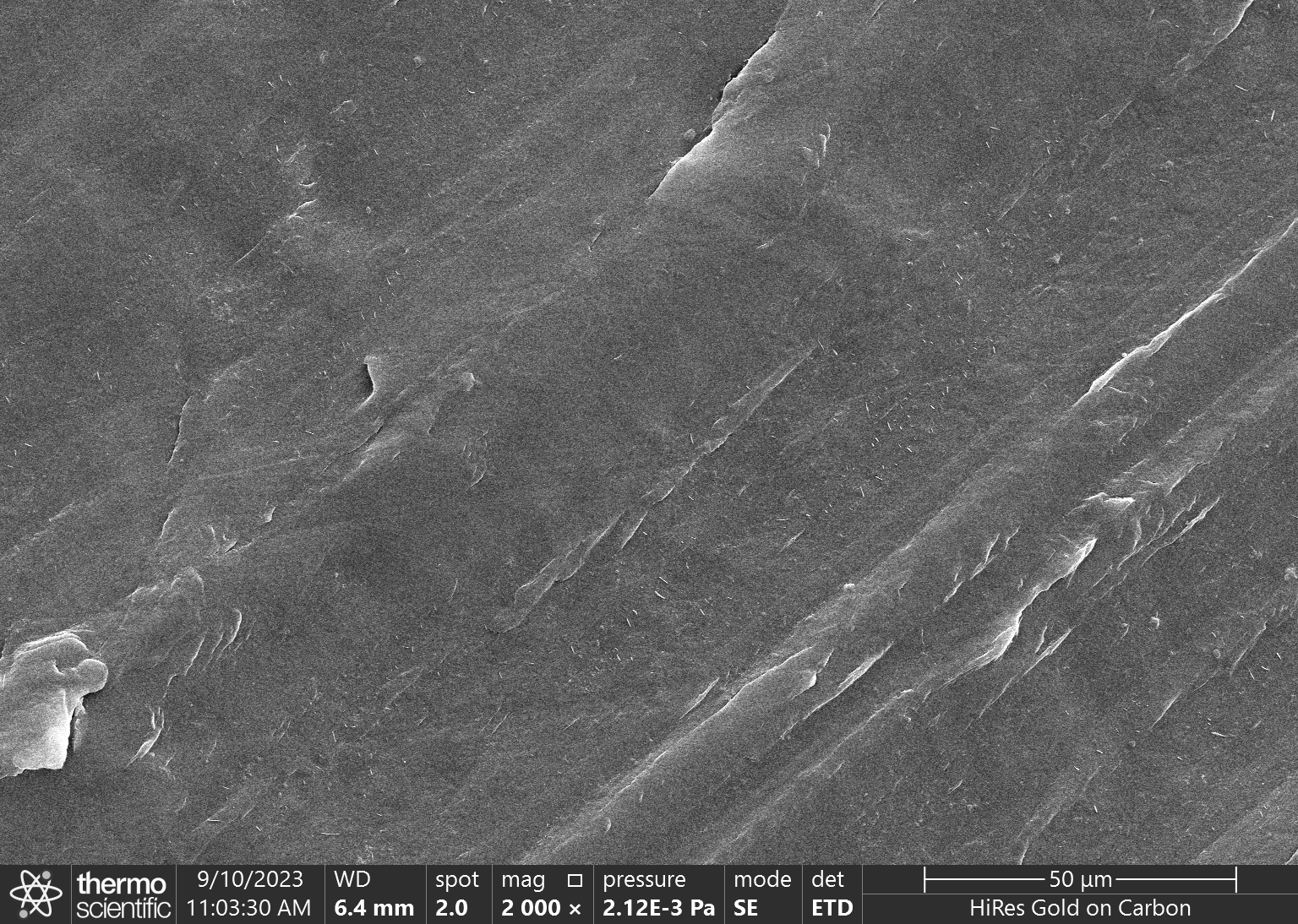

Supplement: Supplementary file 1 — Additional file 1. Original experimental data. [file 13018_2023_4246_MOESM1_ESM.zip › SEM/polyethylene (2).tif]

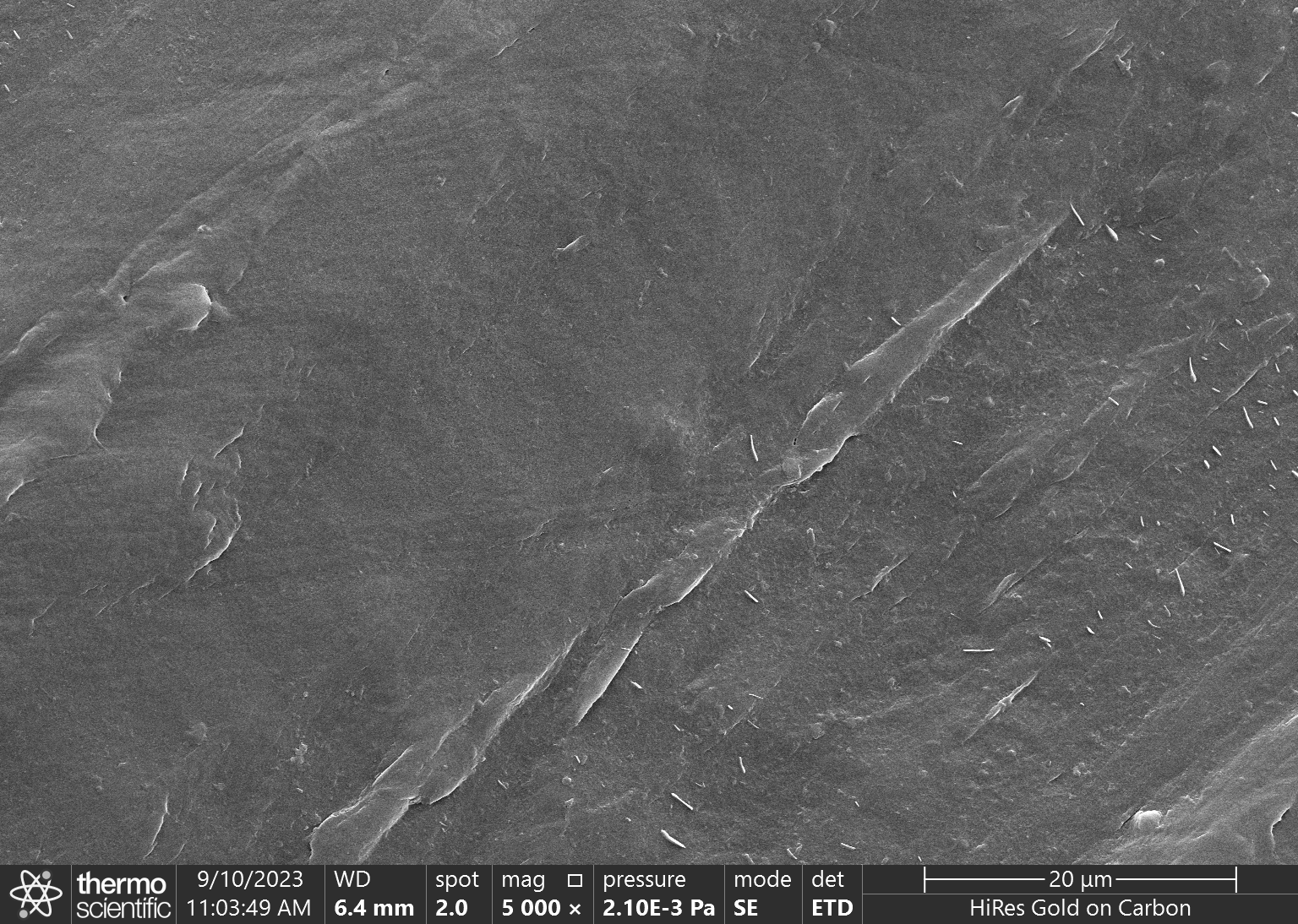

Supplement: Supplementary file 1 — Additional file 1. Original experimental data. [file 13018_2023_4246_MOESM1_ESM.zip › SEM/polyethylene (3).tif]

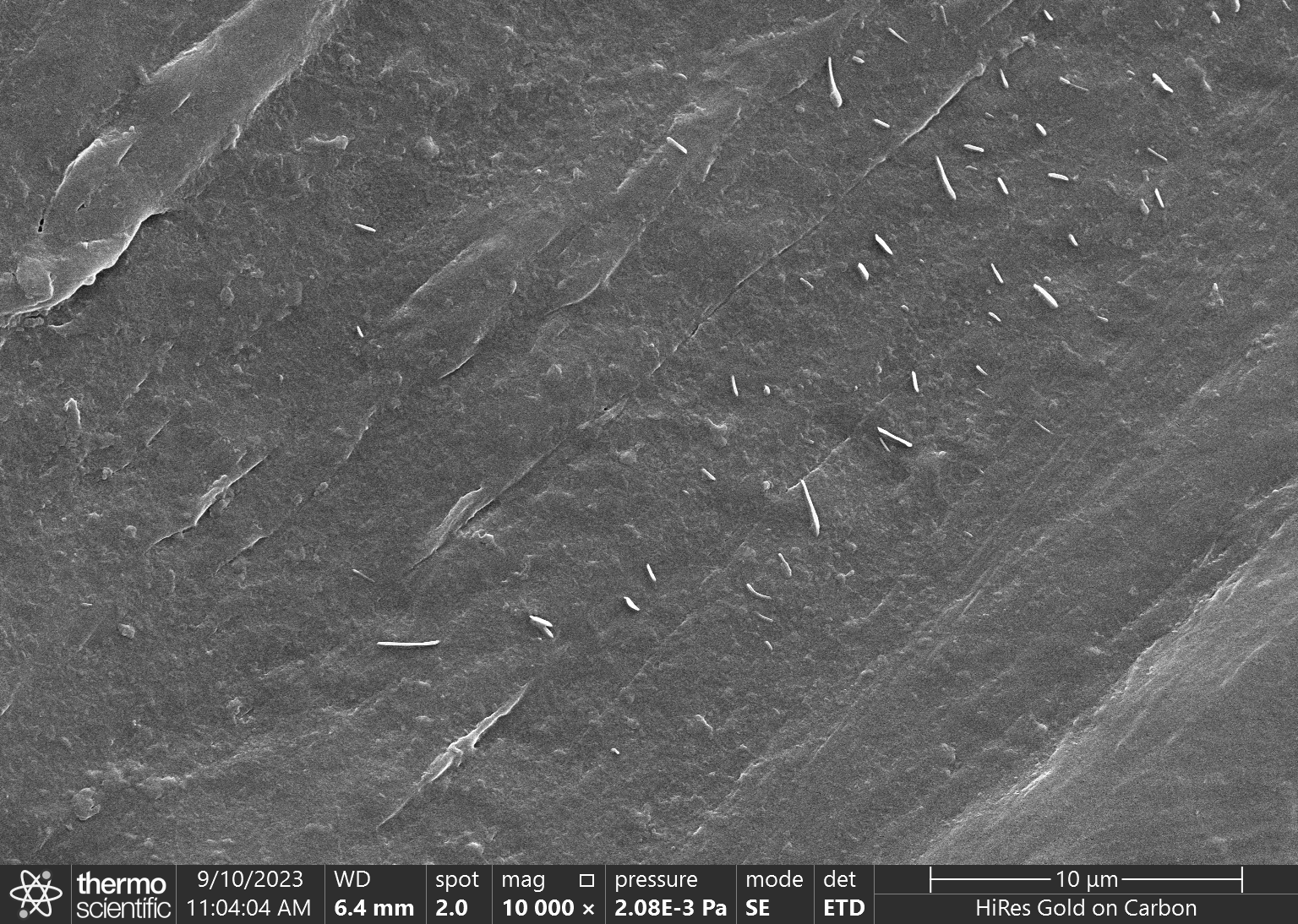

Supplement: Supplementary file 1 — Additional file 1. Original experimental data. [file 13018_2023_4246_MOESM1_ESM.zip › SEM/polyethylene (4).tif]

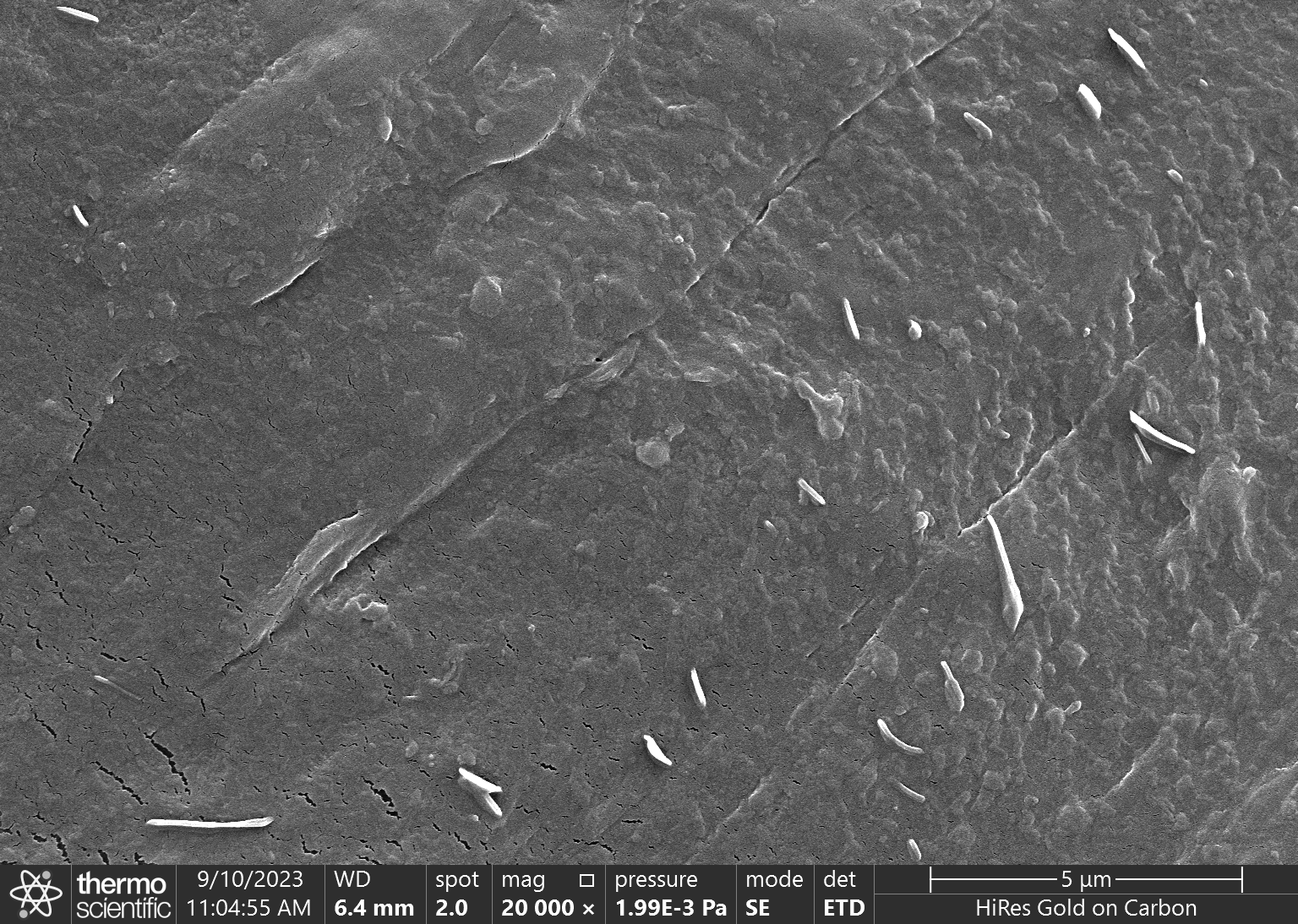

Supplement: Supplementary file 1 — Additional file 1. Original experimental data. [file 13018_2023_4246_MOESM1_ESM.zip › SEM/polyethylene (5).tif]

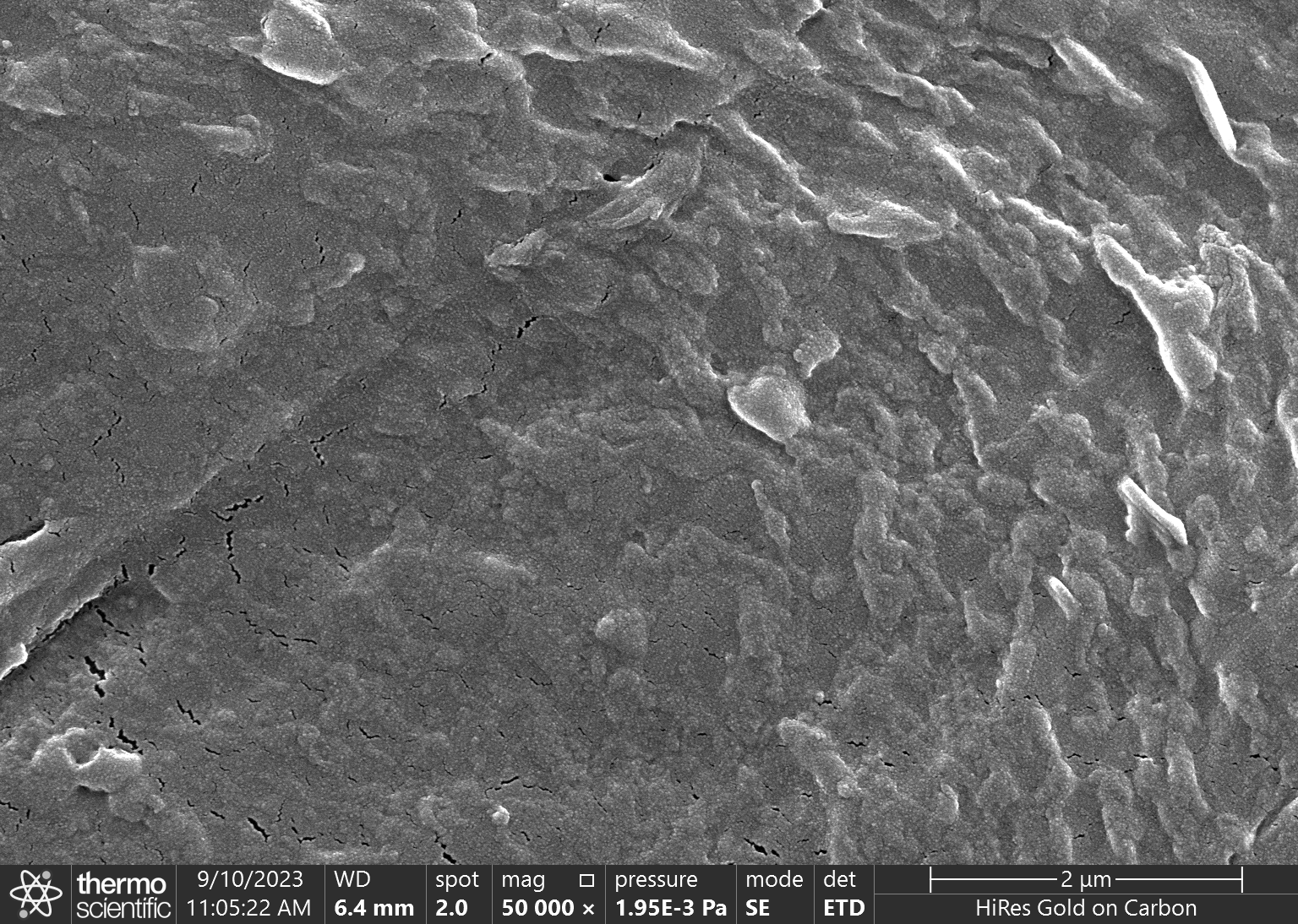

Supplement: Supplementary file 1 — Additional file 1. Original experimental data. [file 13018_2023_4246_MOESM1_ESM.zip › SEM/polyethylene (6).tif]

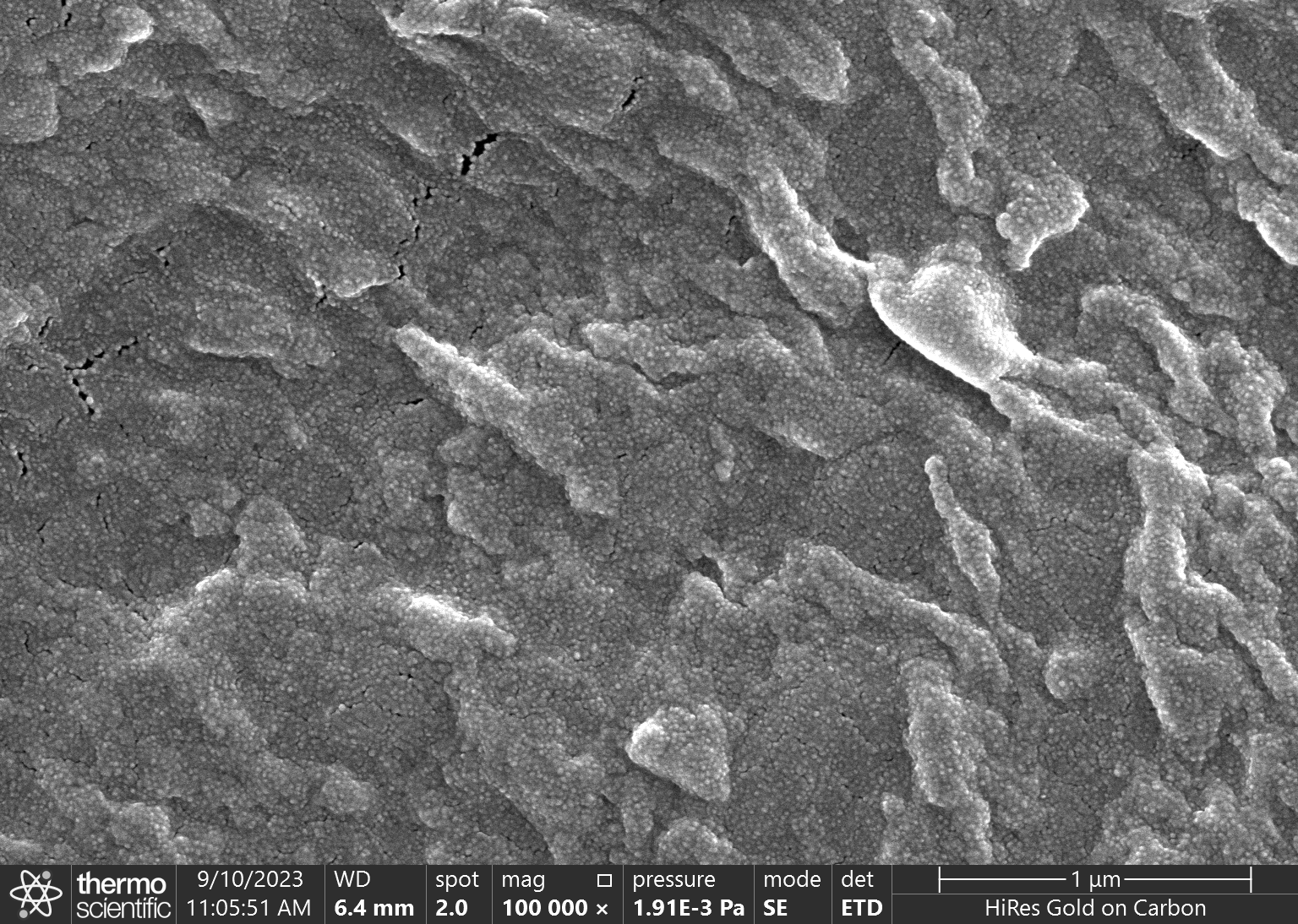

Supplement: Supplementary file 1 — Additional file 1. Original experimental data. [file 13018_2023_4246_MOESM1_ESM.zip › SEM/polyethylene (7).tif]

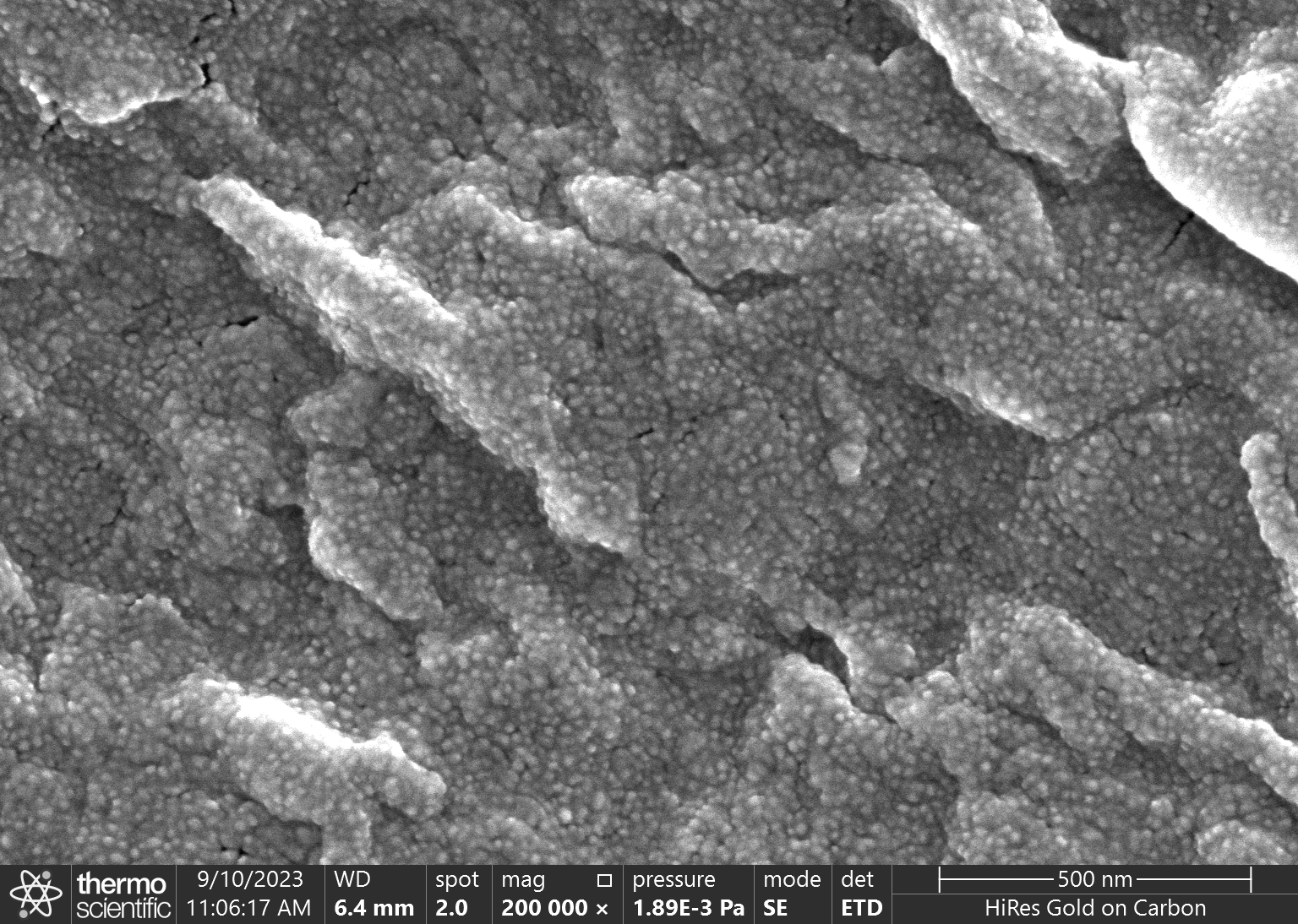

Supplement: Supplementary file 1 — Additional file 1. Original experimental data. [file 13018_2023_4246_MOESM1_ESM.zip › SEM/polyethylene (8).tif]

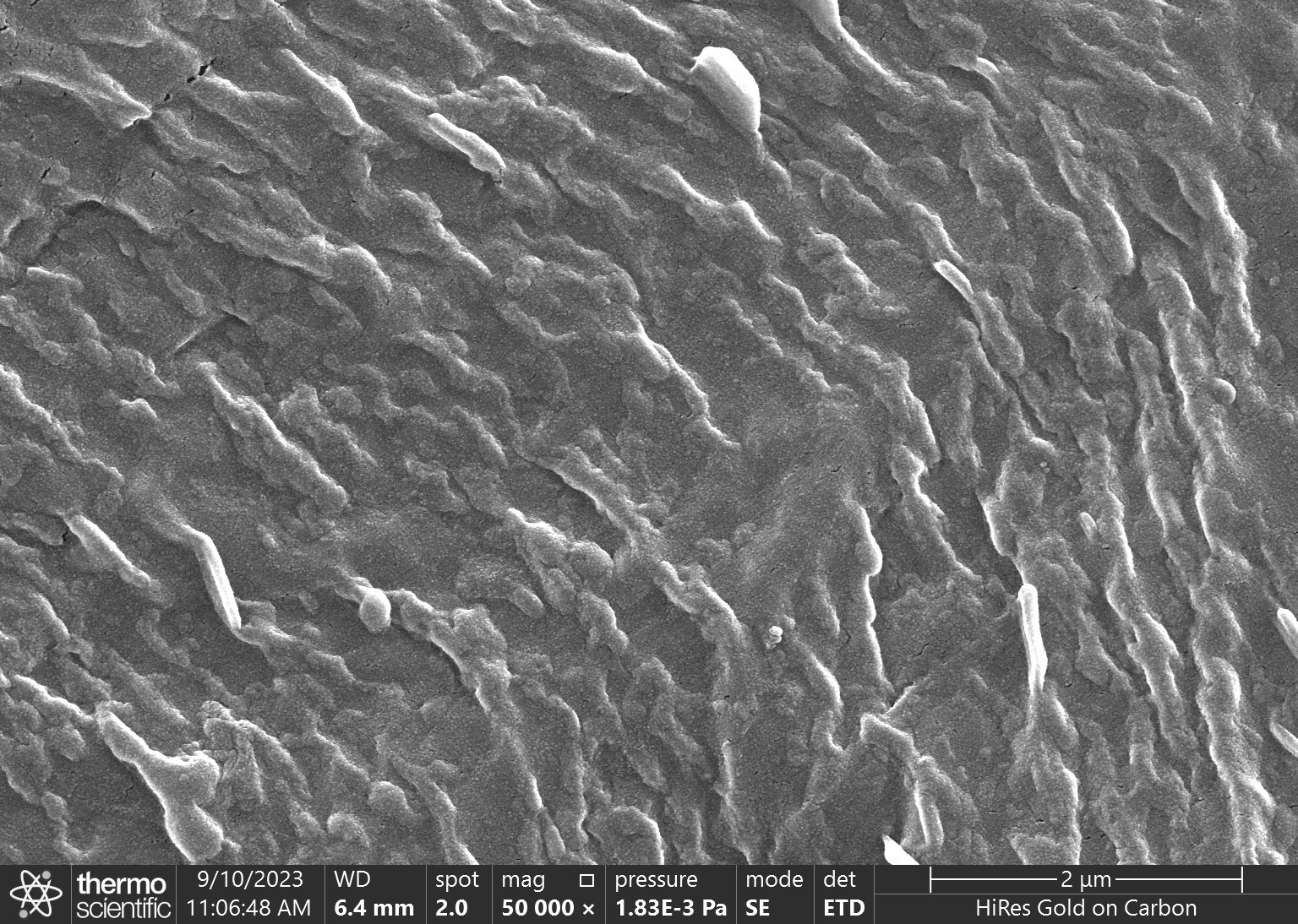

Supplement: Supplementary file 1 — Additional file 1. Original experimental data. [file 13018_2023_4246_MOESM1_ESM.zip › SEM/polyethylene (9).tif]

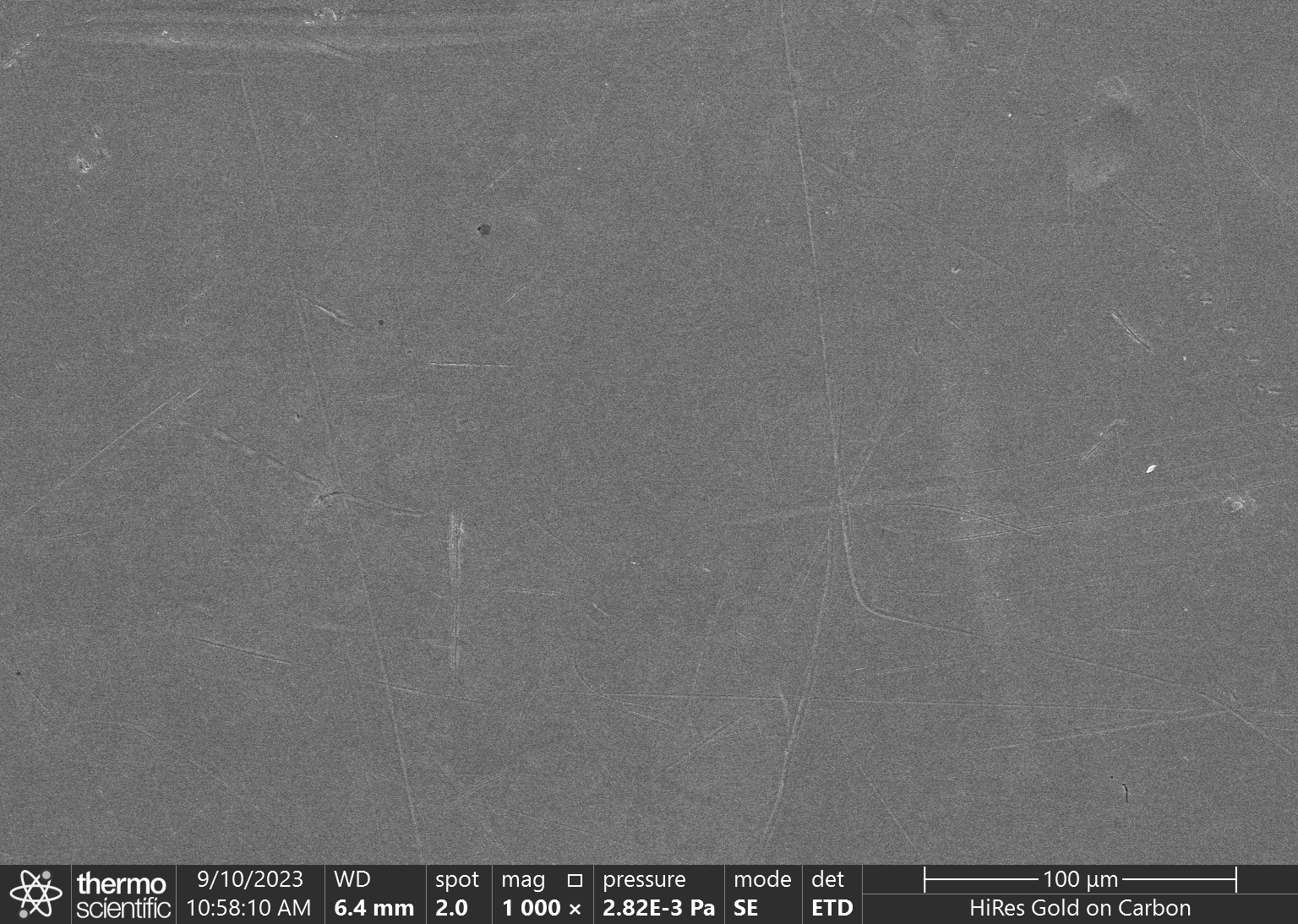

Supplement: Supplementary file 1 — Additional file 1. Original experimental data. [file 13018_2023_4246_MOESM1_ESM.zip › SEM/TA (1).tif]

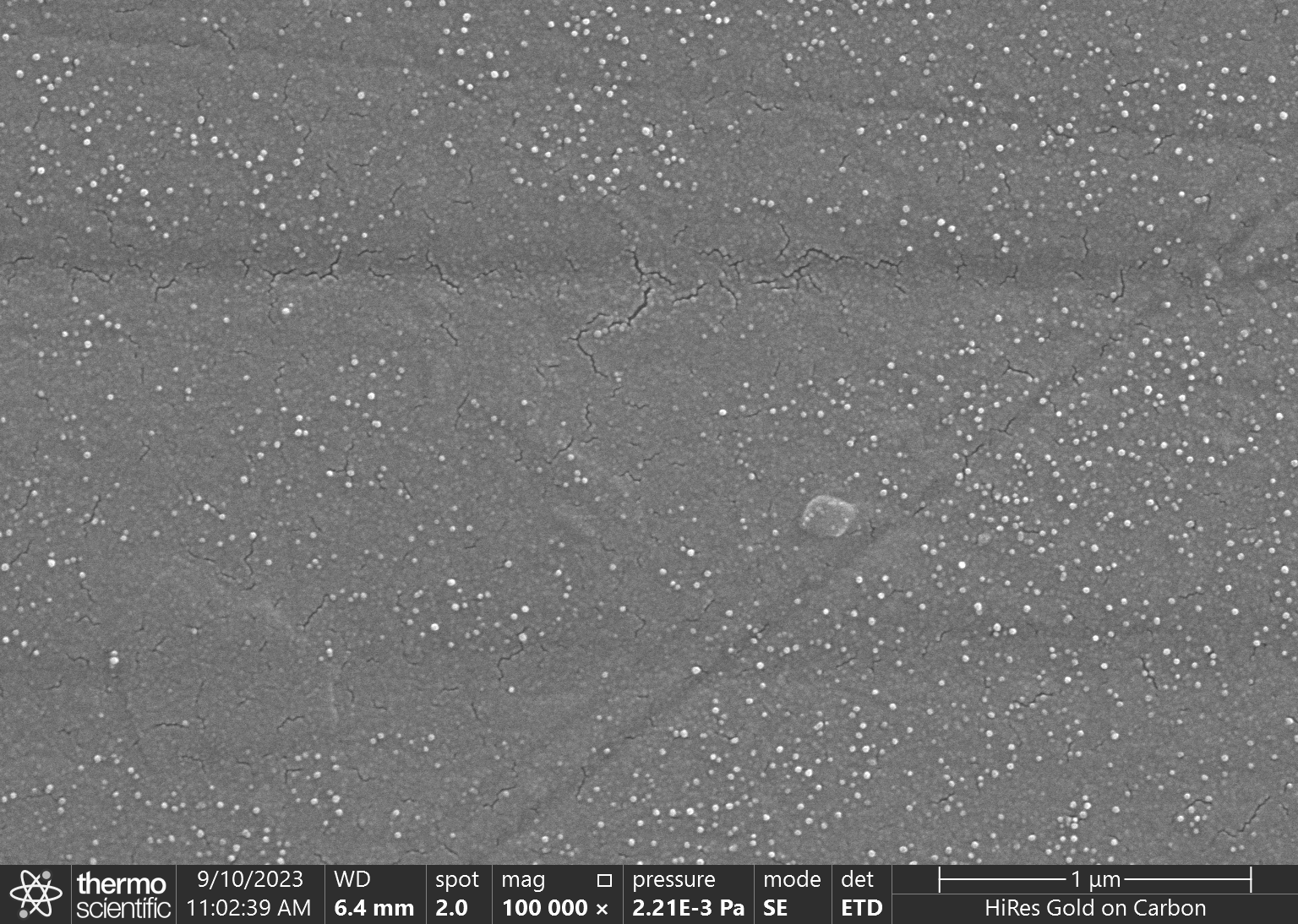

Supplement: Supplementary file 1 — Additional file 1. Original experimental data. [file 13018_2023_4246_MOESM1_ESM.zip › SEM/TA (10).tif]

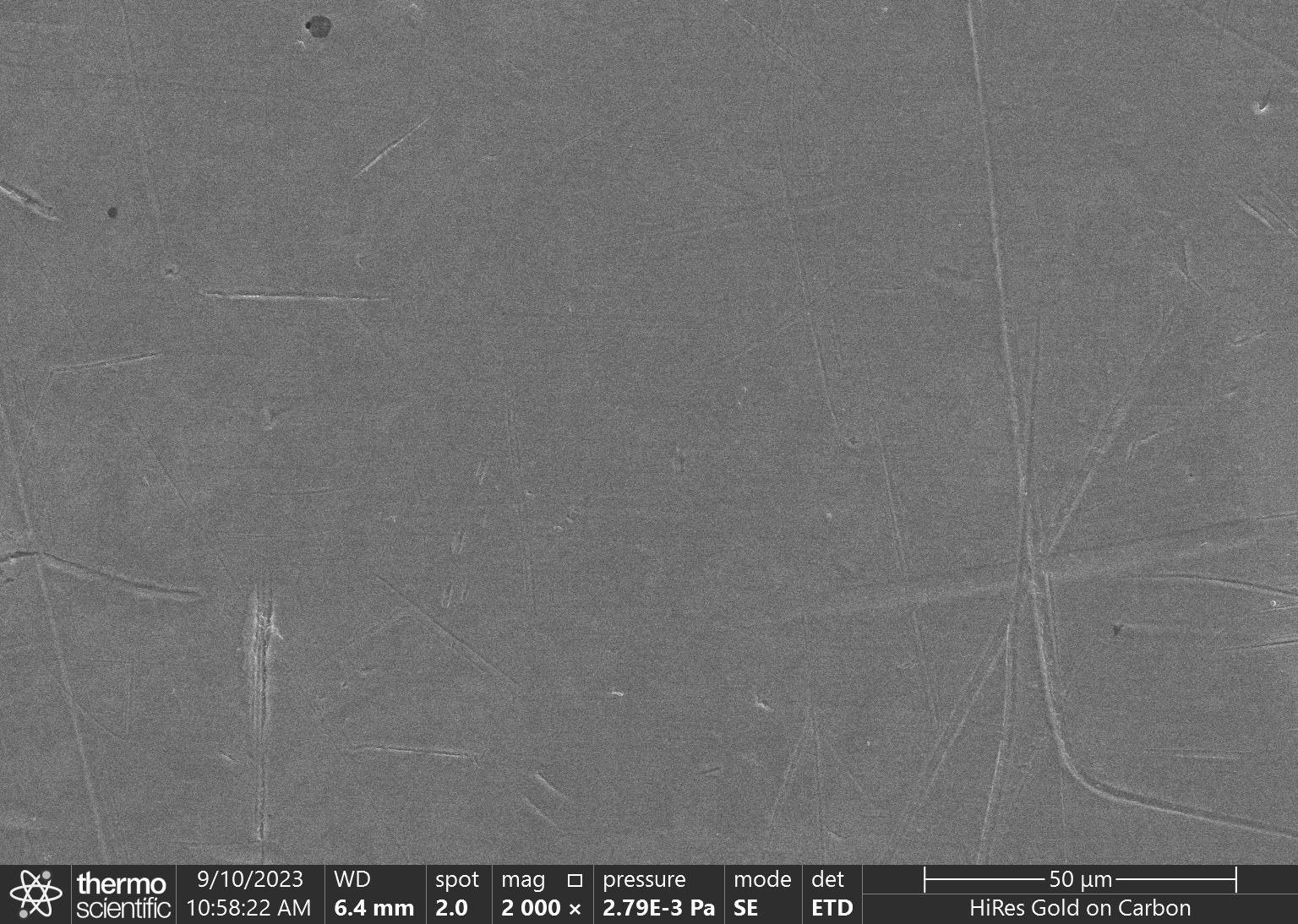

Supplement: Supplementary file 1 — Additional file 1. Original experimental data. [file 13018_2023_4246_MOESM1_ESM.zip › SEM/TA (2).tif]

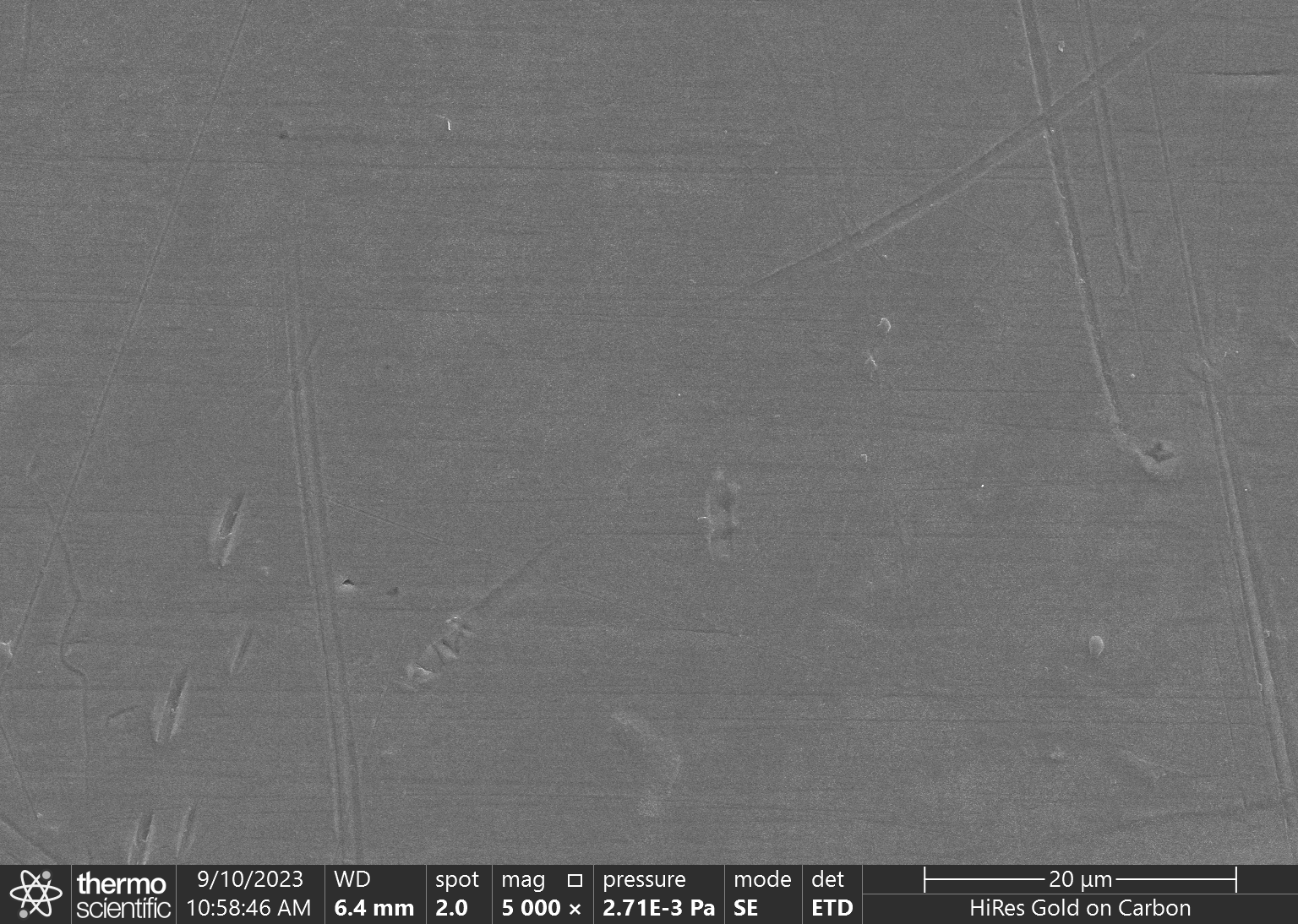

Supplement: Supplementary file 1 — Additional file 1. Original experimental data. [file 13018_2023_4246_MOESM1_ESM.zip › SEM/TA (3).tif]

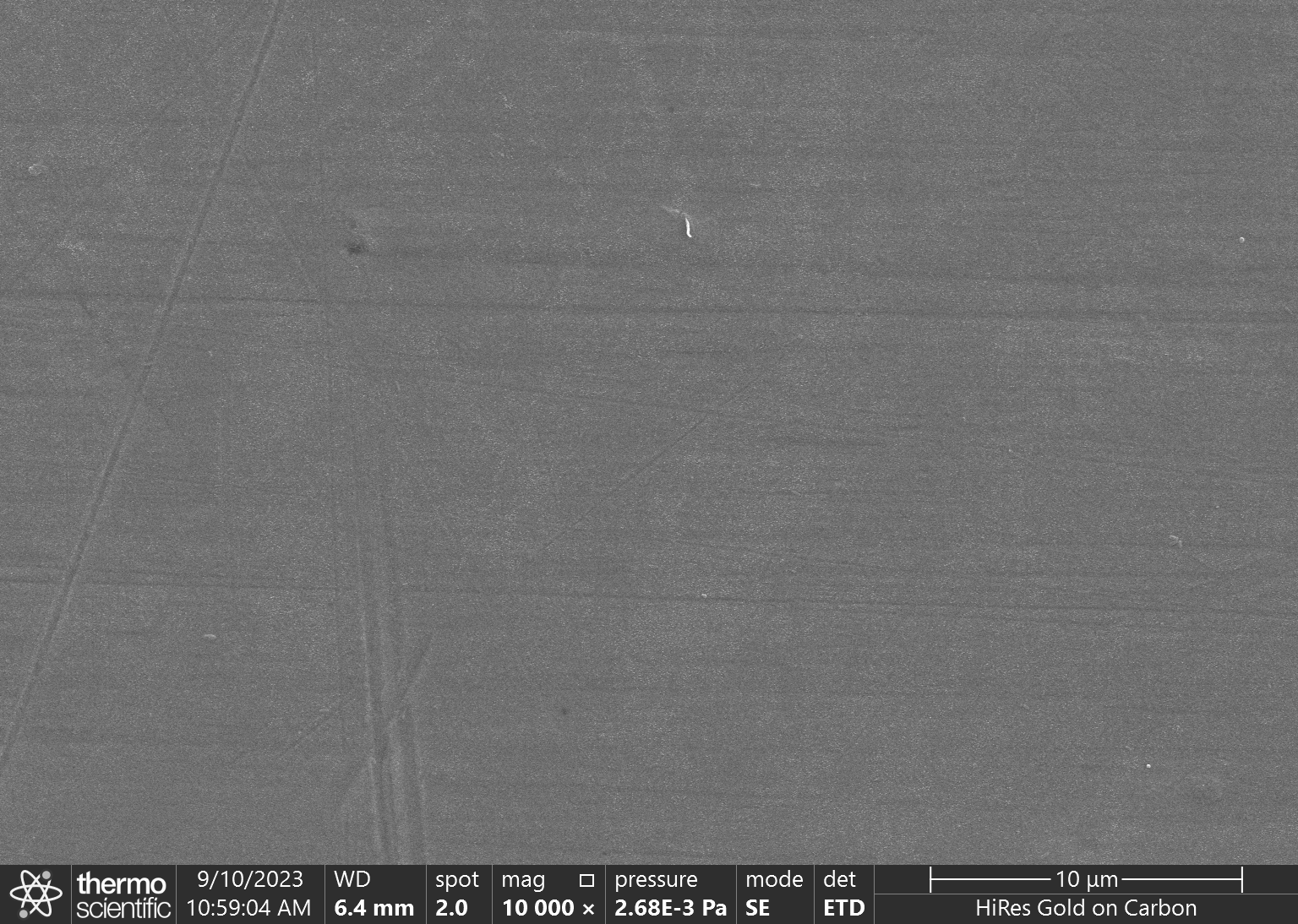

Supplement: Supplementary file 1 — Additional file 1. Original experimental data. [file 13018_2023_4246_MOESM1_ESM.zip › SEM/TA (4).tif]

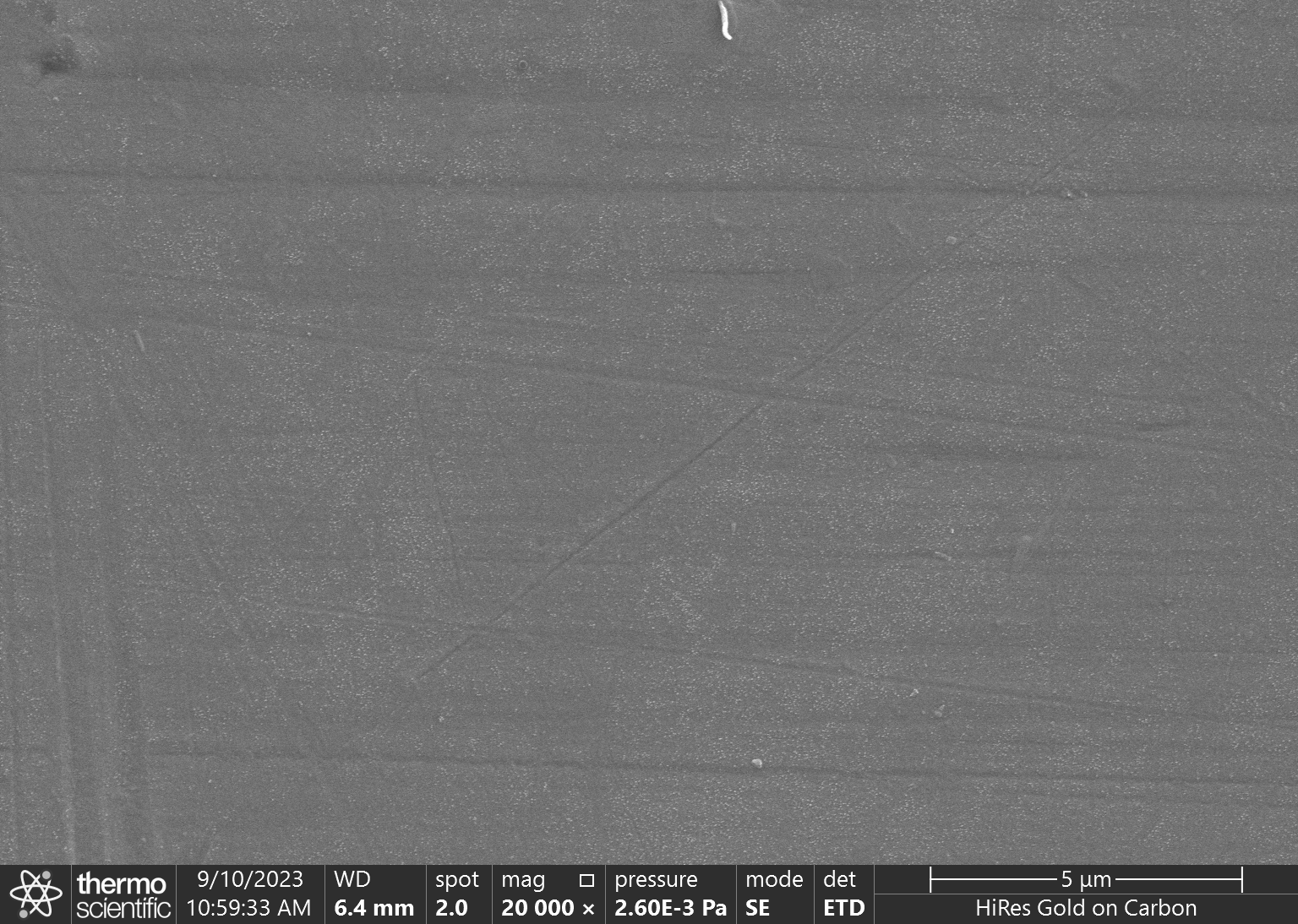

Supplement: Supplementary file 1 — Additional file 1. Original experimental data. [file 13018_2023_4246_MOESM1_ESM.zip › SEM/TA (5).tif]

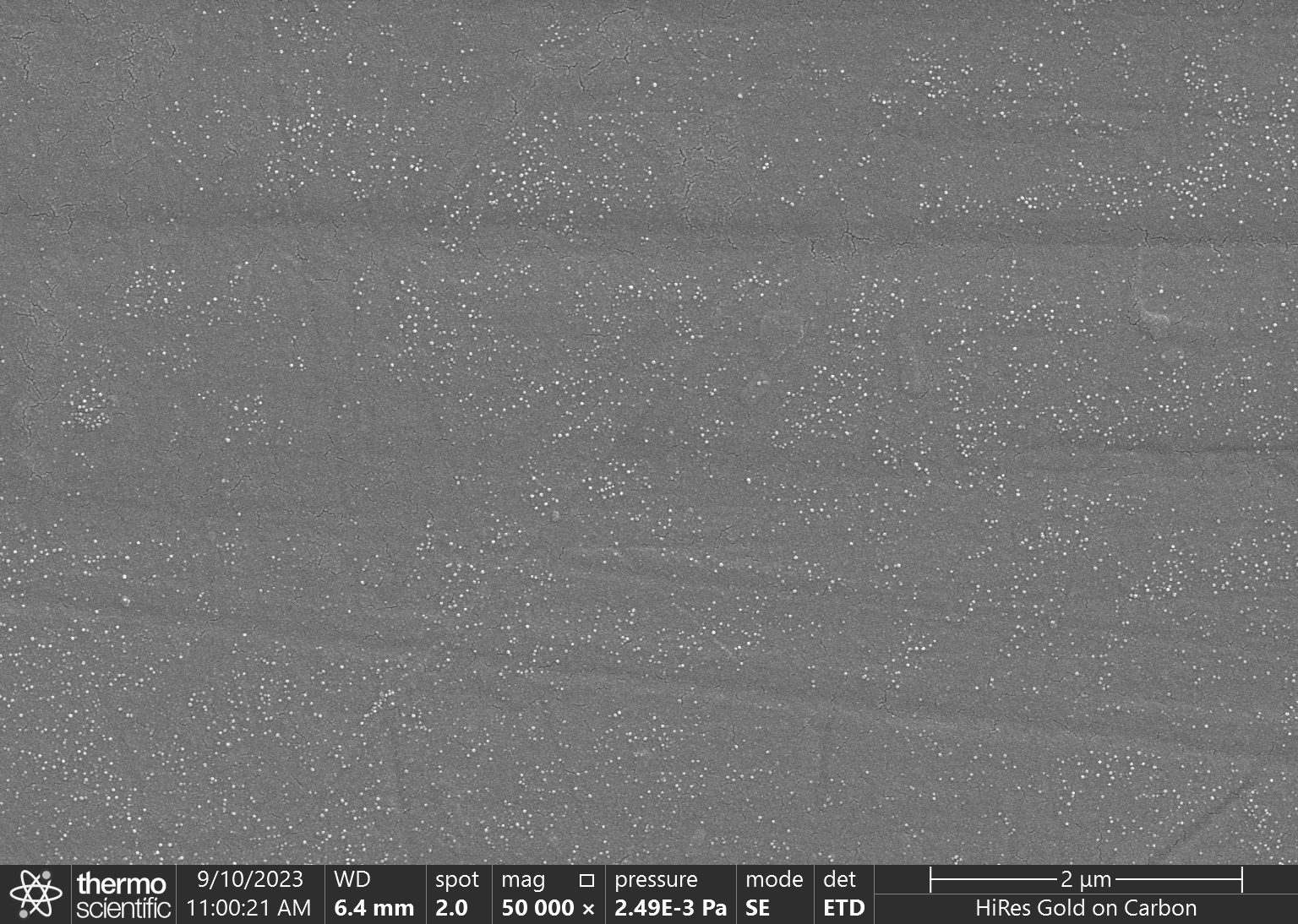

Supplement: Supplementary file 1 — Additional file 1. Original experimental data. [file 13018_2023_4246_MOESM1_ESM.zip › SEM/TA (6).tif]

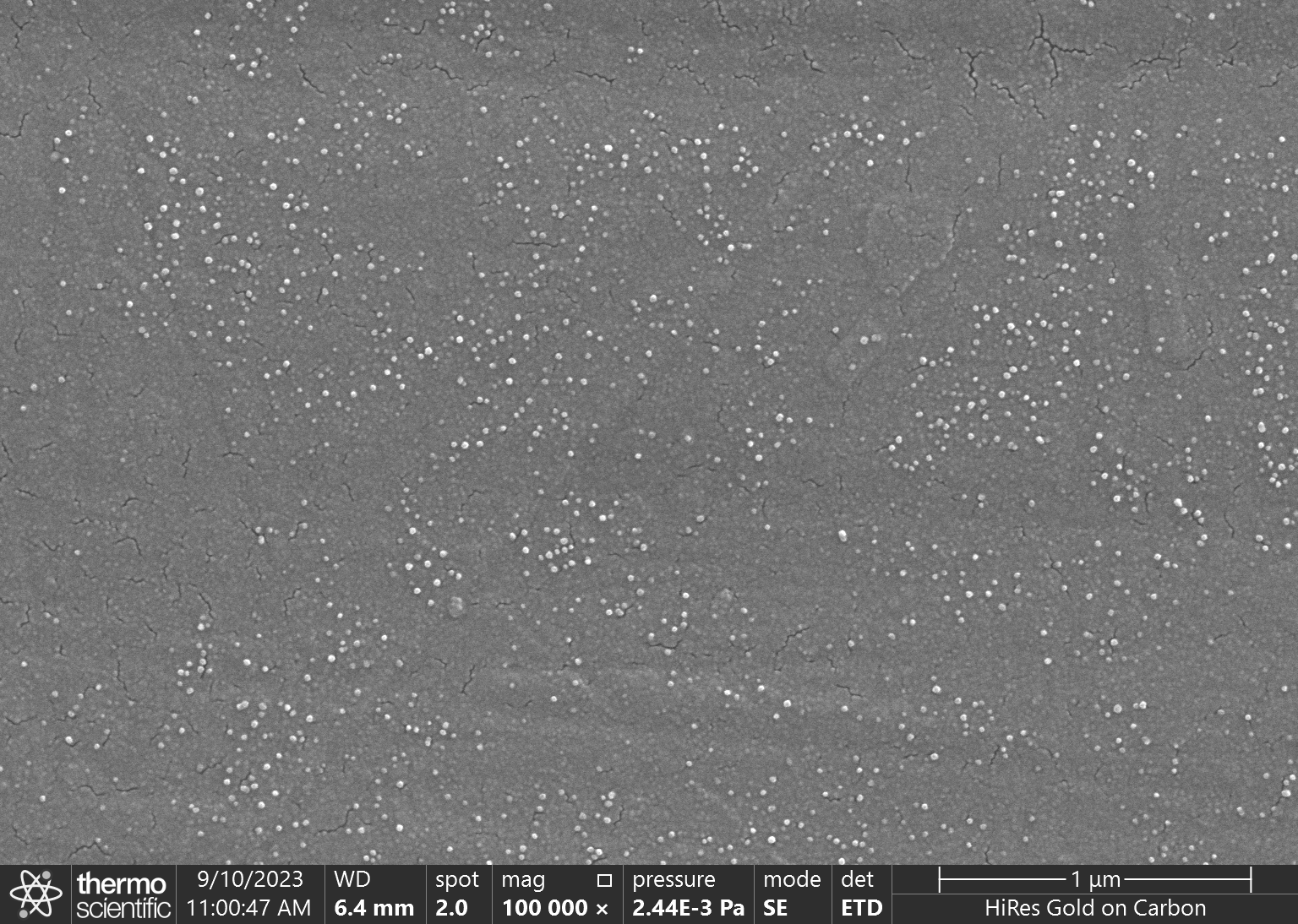

Supplement: Supplementary file 1 — Additional file 1. Original experimental data. [file 13018_2023_4246_MOESM1_ESM.zip › SEM/TA (7).tif]

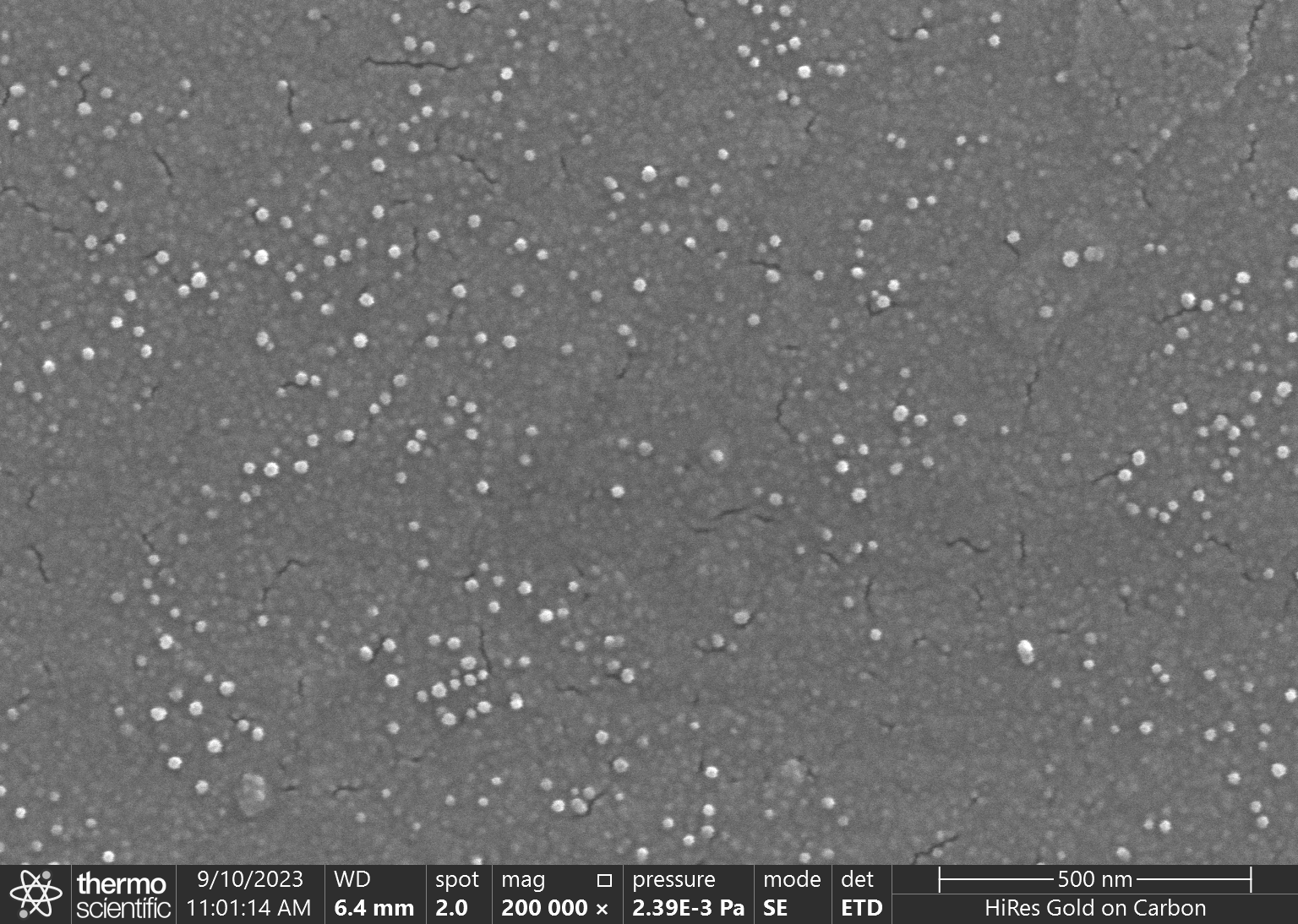

Supplement: Supplementary file 1 — Additional file 1. Original experimental data. [file 13018_2023_4246_MOESM1_ESM.zip › SEM/TA (8).tif]

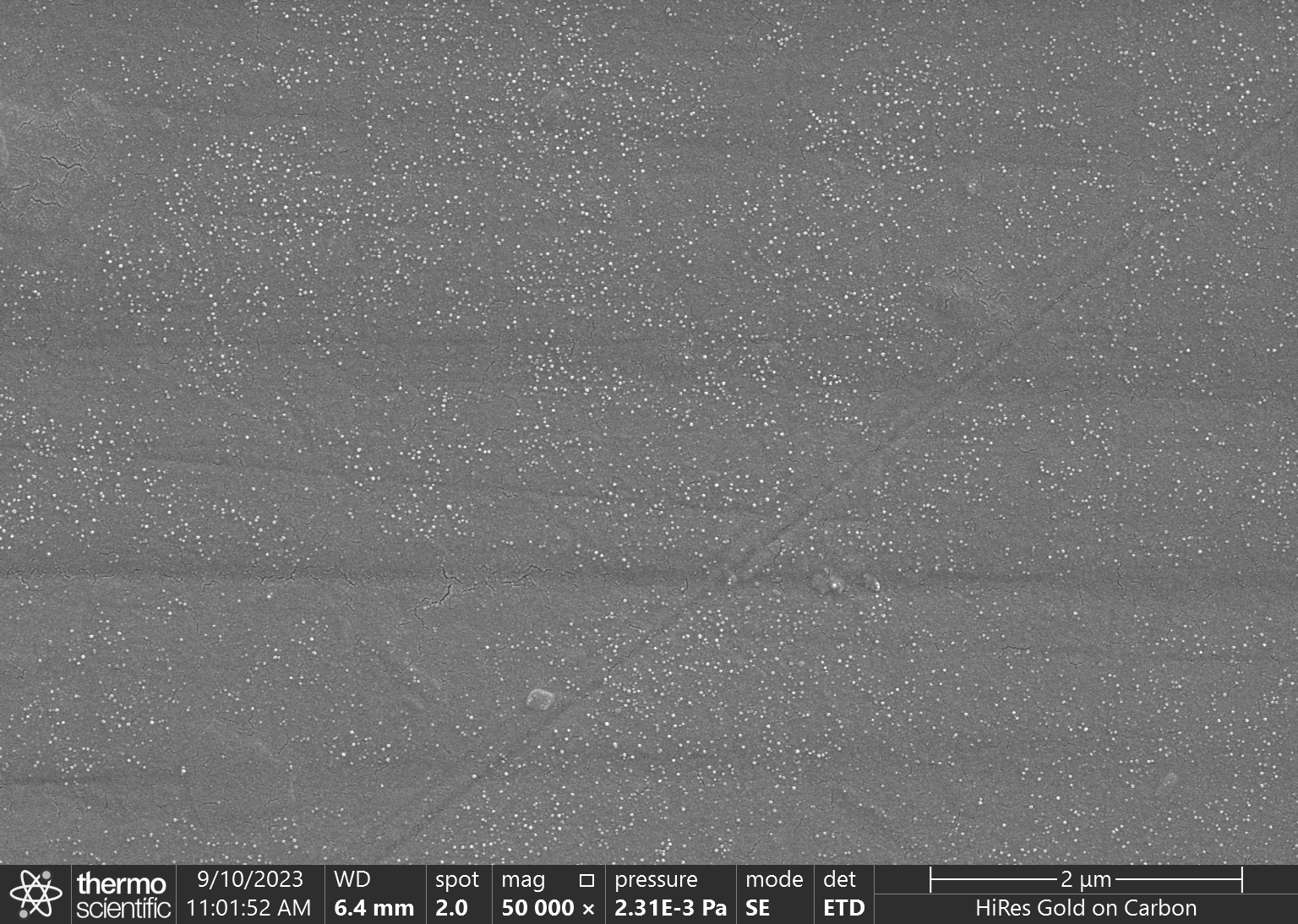

Supplement: Supplementary file 1 — Additional file 1. Original experimental data. [file 13018_2023_4246_MOESM1_ESM.zip › SEM/TA (9).tif]
